# Machine Learning Approach for Fatigue Estimation in Sit-to-Stand Exercise

**DOI:** 10.3390/s21155006

**Published:** 2021-07-23

**Authors:** Andrés Aguirre, Maria J. Pinto, Carlos A. Cifuentes, Oscar Perdomo, Camilo A. R. Díaz, Marcela Múnera

**Affiliations:** 1 Department of Biomedical Engineering, Colombian School of Engineering Julio Garavito, Bogotá 111166, Colombia; andres.aguirre@mail.escuelaing.edu.co (A.A.); maria.pinto@mail.escuelaing.edu.co (M.J.P.); marcela.munera@escuelaing.edu.co (M.M.); 2 School of Medicine and Health Sciences, Universidad del Rosario, Bogotá 111711, Colombia; oscarj.perdomo@urosario.edu.co; 3 Electrical Engineering Department, Federal University of Espirito Santo, Vitoria 29075-910, Brazil; camilo.diaz@ufes.br

**Keywords:** fatigue estimation, Kinect, machine learning, physical exercise, physical rehabilitation, sit-to-stand

## Abstract

Physical exercise (PE) has become an essential tool for different rehabilitation programs. High-intensity exercises (HIEs) have been demonstrated to provide better results in general health conditions, compared with low and moderate-intensity exercises. In this context, monitoring of a patients’ condition is essential to avoid extreme fatigue conditions, which may cause physical and physiological complications. Different methods have been proposed for fatigue estimation, such as: monitoring the subject’s physiological parameters and subjective scales. However, there is still a need for practical procedures that provide an objective estimation, especially for HIEs. In this work, considering that the sit-to-stand (STS) exercise is one of the most implemented in physical rehabilitation, a computational model for estimating fatigue during this exercise is proposed. A study with 60 healthy volunteers was carried out to obtain a data set to develop and evaluate the proposed model. According to the literature, this model estimates three fatigue conditions (low, moderate, and high) by monitoring 32 STS kinematic features and the heart rate from a set of ambulatory sensors (Kinect and Zephyr sensors). Results show that a random forest model composed of 60 sub-classifiers presented an accuracy of 82.5% in the classification task. Moreover, results suggest that the movement of the upper body part is the most relevant feature for fatigue estimation. Movements of the lower body and the heart rate also contribute to essential information for identifying the fatigue condition. This work presents a promising tool for physical rehabilitation.

## 1. Introduction

Physical exercise (PE) is defined as any activity performed by the muscles that requires more energy than a resting state [1]. According to the World Health Organization, PE is a fundamental tool to prevent and treat many non-communicable diseases [2], such as cardiovascular diseases, cancer, stroke, and diabetes. Therefore, to help patients and clinical staff to achieve specific rehabilitation aims, PE has been incorporated into different rehabilitation programs [3,4]. On the one hand, PE is used for improving the patient’s cardiovascular and respiratory capabilities in cardiac [5,6,7] and pulmonary [8,9] rehabilitation sessions. Furthermore, in oncology rehabilitation, the PE helps mitigate the pathological fatigue effects [10,11], which is a common symptom presented in patients with cancer [12]. This means that patients are easily exhausted when performing activities of daily living [13]. In addition, the PE is implemented in neuromuscular [14,15,16] and musculoskeletal rehabilitation [17,18] to restore joint mobility and muscle strength of affected limbs.

The main aim of PE in rehabilitation is to develop the health-related physical fitness (HRPF) state of the patients [3], which refers to the components that are required to have a healthy life. In general, these components are focused on preventing illness or improving functional health, instead of working sports performance [19]. The HRPF capabilities can be divided into three individual groups [3,19]: body composition, which considers the distribution of the different body tissues (water, fat, muscle, and bone) [19]; musculoskeletal, which refers to the strength, endurance, power and flexibility of the muscles [20]; and cardiorespiratory or aerobic capability, related to the ability of the circulatory and pulmonary systems to provide oxygen for creating energy during long periods of activity. The cardiorespiratory capabilities are usually assessed with the maximal oxygen uptake ($VO_{2MAX}$) [21]. Nevertheless, studies have shown that the anaerobic capability is also relevant for a good quality of life because it focuses on the body’s ability to produce energy without oxygen, which is the metabolic way used for sudden movements with a short duration, commonly executed in daily life [3,20,22,23].

Essentially, stretching, endurance and resistance exercises with external loads or human body weight are used for developing the musculoskeletal group [20]. On the other hand, aerobic activities (e.g., walking, jogging or riding) are implemented for the cardiorespiratory elements [19]. Finally, the anaerobic capability is amplified by short-duration powerful activities (e.g., vertical jumps, sit to stand or running short distances) [22]. Therefore, various activities can be implemented to achieve different goals [3].

Despite the benefits of PE, several considerations must be kept in mind for its implementation in rehabilitation, because taking patients to extreme exercise conditions and high fatigue states might lead them to suffer physical or physiological complications [24]. Considering this, it is required to design a personalized exercise plan at the moment to use PE as a clinical tool and achieve the different objectives of each rehabilitation program. This plan must be prescribed by a specialized health care professional, according to the unique conditions of each patient (e.g., weight, height, age, injuries, medication, pathologies [25]). Commonly, the training plan considers the activities, frequency, time, and intensity of training [25]. Studies have shown that the intensity is the most relevant feature at prescribing PE [26,27] because it determines the amount of energy expenditure and can be seen as the “dose” of the prescription [3]. Moreover, it is used to allocate the exercises in three groups: low-intensity exercises (LIEs), moderate-intensity exercises (MIEs) and high-intensity exercises (HIEs) [3,23].

The LIEs are composed of soft activities that demand a low energy cost (e.g., slow walking on a flat surface), and it is used for patients with extreme risk conditions [3]. The MIEs contemplate non-stopped activities with a long duration that require a low effort (e.g., walking on a slope between 20 to 60 min) [3]. At first, the LIEs and MIEs were the only classes implemented in rehabilitation, especially in cardiac rehabilitation because they let the clinical staff manage the intensity easily and had shown to be sufficient to reduce chronic disease risk factors [28,29]. In contrast, HIEs are forceful activities with a short duration (between 15 s to 5 min, depending on the intensity), which can be divided into recovering and training periods [30]. Since several studies have demonstrated that HIE is more effective at increasing the $VO_{2MAX}$ [20,21,22,28,31,32,33,34,35,36], this training technique has been widely used in physical rehabilitation [37]. Furthermore, the American Heart Association has incorporated the HIE into its recommendation manual for patients with heart diseases [38]. Nevertheless, the infinite interval variations and the difficulties at managing the intensity make the HIEs prescription a complex task [23].

Although many activities can be used as HIEs; it is highly recommended to perform the exercises that reflect daily life motions (e.g., jumping, carrying loads, or climbing stairs). In general, because they elicit the metabolic ways and the muscular groups required for a healthy life [22]. Moreover, they are easy to implement in clinical scenarios [22]. Thus, bearing in mind that sitting and standing are some of the most common activities, the sit-to-stand (STS) test is widely implemented in physical rehabilitation [39]. This test consists of sitting and standing from a chair as fast as possible during a determined period (between 30 to 120 s), and it is considered as one of the hardest exercises [40]. Therefore, studies have demonstrated that it is indispensable for increasing $VO_{2MAX}$ and assessing the patients’ physical state [39,41]. However, due to its high intensity, it requires special monitoring compared to the other HIEs [42].

Hence, considering the importance of the STS test in the rehabilitation programs and the risks of taking patients to high fatigue conditions during sessions, there is a need to develop methods that allow managing the exercise intensity [42].

### Exercise Intensity and Fatigue Regulation Background

Intensity can be defined in two ways: absolute, which refers to the complete quantity of energy used during the whole training, and relative, which contemplates the rate of energy implemented in the activity [43]. Hence, the more energy required for an activity, the harder it will be. Because the relative intensity allows obtaining a real-time metric, it is the most used in physical rehabilitation sessions for monitoring the patient’s condition [43]. However, quantifying the amount of metabolic energy expended is a complex task due to the fact that the human body executes too many jobs at the same time and has different ways of producing energy [3]. Therefore, several techniques have been explored to get an indirect estimation of the exercise intensity.

One approximation consists of estimating the relative intensity by using the metabolic equivalent (MET). It is a unit that represents the relation between the rate of energy expended in physical activity and the rate of energy expended in a resting state, commonly measured in Kcal×Kg${}^{-1}$×$h^{-1}$ [44]. In this way, the LIE is lower than two METs, the MIE is between two and six METs, and the HIE is higher than six METs [45]. Obtaining the rate of energy expended is not an easy task. Therefore, many exercises have been classified by global organizations according to some standards of healthy people [46]. However, this implementation of the MET unit has been widely criticized for the exercise intensity regulation in rehabilitation sessions. It is because it does not allow monitoring the patient’s condition during the session [47]. Hence, it is more commonly used to get a general idea about the type of exercise implemented in the training plan [44].

As continuous monitoring is essential for patients with chronic diseases during physical sessions, it is preferred to estimate the exercise intensity based on metrics obtained directly from the patients [3]. Thus, other methods that consist of measuring physiological parameters related to the energy expended have been proposed—for example, monitoring the patient’s breathing rate, blood lactate level, oxygen saturation, or blood pressure [48,49,50].

The oxygen uptake (VO${}_{2}$, usually measured in mL/min/kg) is considered one of the best ways to determine the exercise intensity, because of its linear relationship with the energy cost [51]. Furthermore, the VO${}_{2}$ can be changed easily to METs units [52]. Nevertheless, it requires complex instrumentation, and it is difficult to measure directly. Hence, health professionals prefer not to apply this technique in their sessions [3]. A similar case is presented for the blood lactate level, blood pressure, and oxygen saturation, where a static position is required to obtain reliable measurements [3].

On the other hand, the heart rate (HR) can be easily estimated during exercise and has shown a linear relation with the VO${}_{2}$ [50]. Therefore, HR is the most used physiological parameter in physical rehabilitation, especially for aerobic training [50]. Bearing in mind that each person may present different HR values during resting or training conditions, it is better to implement the heart rate reserve (HRR) for intensity monitoring [3].

The HRR represents the safe range of person HR values, calculated by the difference between the maximum HR (estimated by exercise test or the age) and the resting HR of a person [3]. This metric can be provided as a percentage, which represents the part of the range that is being covered. Hence, a value of 100% means that the person reaches his/her maximum HR. In contrast, 0% means that the person is at his/her resting HR. It can be implemented to divide the exercise intensities in such a way: the LIE is between 20–39%, the MIE is between 40–59%, and the HIE is between 60–84% of the HRR [3]. Nevertheless, studies have shown that the HR stops rising linearly when reaching its maximum values and loses its relationship to the VO${}_{2}$. Thus, it is not recommended to only use this indicator for monitoring HIE [50].

Other methods consist in estimating the patient’s fatigue because it is understood as a lack of energy to keep performing an activity [53]. Fatigue has been considered as a subjective experience [54], which describes a decrease in physical performance associated with an increase in a task or exercise’s real/perceived difficulty [55].

Electromyography (EMG) is considered the gold standard to detect muscular fatigue because it directly measures the bio-electrical function of the muscles [56,57]. However, the EMG processing is also a complex task to execute in real-time, since it requires power and frequency analysis [58]. It is needed to consider the noise generated by external factors and the location of the electrodes, especially as they are not always going to be placed in the same position [59]. Furthermore, some exercises implement many muscular groups, which makes it necessary to use several electrodes [59].

Other techniques implement subjective methods where the patients are asked about the level of perceived exertion or fatigue, according to established ordinal numeric scales [60]. The ten points Borg’s scale (Borg CR10) is composed of 11 levels (from 0 to 10), and it is one of the most applied in physical rehabilitation [61]. On this scale, the LIE corresponds to 0–3, the MIE corresponds to 4–6, and the HIE corresponds to 7–9 values, considering that the ten value is the maximum effort and means that the patient is not able to continue with the exercise [3]. Despite its ease of application, studies have demonstrated that, due to its subjectivity, it does not always represent the real intensity compared to the physiological parameters (specifically, to the VO${}_{2}$) [62]. Finally, the last technique is based on the idea that fatigue can be seen as a decrease in the performance of the user [63]. Current studies have shown that some temporal, kinematic, and dynamic features of the activity executed may change according to the exhaustion level of the user [64,65,66]. In general, ambulatory sensors are used to estimate the related performance features (e.g., accelerometers, gyroscopes, pressure sensors, and force platforms) because they allow measuring of easily physical metrics of the user in real scenarios [67,68]. Furthermore, computer models have been developed through the application of machine learning techniques to estimate whether the user is in a fatigued or non-fatigued state, i.e., only two states of fatigue. Studies have proposed models for different exercises, such as vertical jump [69], lower limb endurance training [66], and walking [70], showing an accuracy between 85% and 95%.

Although this novel technique presents a significant potential for clinical scenarios because it provides an objective indicator of the user’s fatigue condition [70], having only the estimation of two fatigue states limits more accurate monitoring of the user’s performance during therapy. However, these systems implement sensors that are easy to adapt and use in rehabilitation environments, providing a practical and useful tool for the health staff [67,68]. Moreover, due to the global health emergency caused by the coronavirus disease 2019 (COVID19), the need for home clinical tools has increased lately [71]. Therefore, this type of technology presents a significant potential for telemedicine in rehabilitation applications. Nevertheless, this method is highly dependent on the exercise type, and each activity performance is assessed with different features [72], which requires adapting the whole system to the corresponding activity.

Regarding the STS test, to the author’s knowledge, two studies have explored this novel technique for this exercise. Aguirre et al. [65] determined which STS features present a relation with fatigue, implementing a Kinect depth sensor and the Borg’s scale, with twenty healthy volunteers. Results showed that two temporal and three kinematic STS features present a significant lineal relation to the exhaustion level. However, a model to estimate fatigue is not developed.

Otherwise, Jiménez et al. [42] presented a case of study for detecting fatigue employing EMG signals and a smartphone accelerometer, with an obese and sedentary volunteer who performed eight STS tests. Results exhibit that relative energy acceleration of the movement increase, and the number of repetitions decreases when the person is physically exhausted. Nevertheless, an estimation model is not displayed, and it is concluded that future work should use these characteristics to develop robust models [42].

We consider the advantages and disadvantages regarding the novel fatigue estimation method based on a decrease in the performance of the user and machine learning models exposed above. Specifically, nowadays, the novel methods of machine learning developed only consider two states of fatigue, i.e., fatigued or non-fatigued state, which limits more accurate monitoring of the user’s exhaustion during therapy and thus, determines the possibility of improving the user’s performance during therapy. We also consider the relevant information of the HR about exercise intensity, the importance of monitoring patients’ fatigue condition during exercise to avoid any injuries or affect the rehabilitation process, and the wide use of STS exercise in physical rehabilitation. This work aims to carry out a study with 60 healthy volunteers to develop and evaluate a machine learning model based on the evaluation of the participant’s exercise performance to classify three fatigue levels (low, medium, and high) to be more specific in regards to user’s fatigue state monitoring in STS exercise. For this purpose, the HR of the participants in the execution of the STS exercise was monitored, and kinematic and temporal characteristics of the movement were obtained through the Kinect. This device was chosen for its ease of adaptation and use in rehabilitation settings, providing a practical and helpful tool for healthcare personnel.

## 2. Materials and Methods

Bearing in mind the motivation and the related works mentioned in Section 1, this section presents the methodology applied to develop the proposed model. In general, the model estimates three levels of fatigue in the STS exercise by monitoring 32 kinematic/temporal features and the user’s heart rate. To this end, this model is based on machine learning techniques, developed with 660 STS registers obtained from 60 healthy people and Borg’s scale. Therefore, the first step consisted of an experimental study with 60 healthy participants to obtain the corresponding data to develop and assess the proposed fatigue estimation model.

### 2.1. Subjects Recruitment

A total of 30 females and 30 males were recruited to perform a 2 min sit-to-stand test, according to the following criteria:

*Inclusion criteria:* Healthy adult subjects between 18 and 30 years old and with a weight between 50 and 75 Kg were considered. Furthermore, volunteers must have been in a non-fatigued condition, according to the “multi-dimensional fatigue inventory”. This tool is a 20-item questionnaire used for measuring the user’s fatigue condition, according to 5 different classifications: general fatigue, physical fatigue, mental fatigue, reduced motivation, and reduced activity.

Exclusion criteria: subjects with physical impairments that prevent them from sitting down and standing up, cognitive impairments that do not allow them to follow instructions, conditions that put them at risk in a fatigued state and use prostheses or orthoses in their limbs were excluded from the study.

Finally, the volunteers signed informed consent to clarify that they voluntarily accepted to participate in this study. The ethics committee accepted this protocol of the university “Colombian School of Engineering Julio Garavito” (Bogota, Colombia). The mean and standard deviation (M ± SD) of volunteers’ demographic data for the female and male groups are shown in Table 1.

### 2.2. Materials

**Figure 1:**
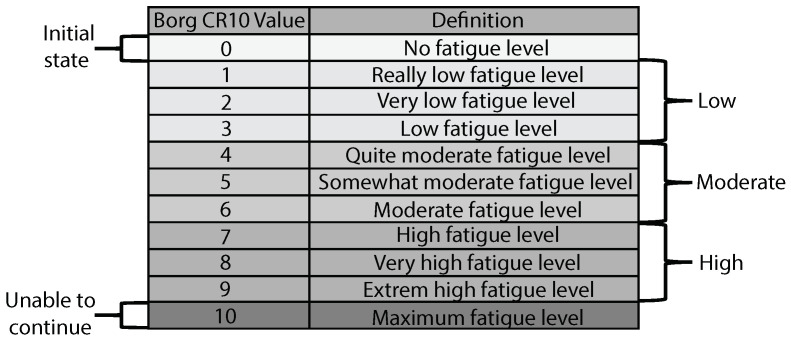
Borg CR10 definition table.

To analyze the STS kinematic, the heart rate and the fatigue level of each volunteer, a multitasking application (i.e., a multi-wire application not affected by the sampling rate of each sensor utilized) was developed to incorporate and synchronize the following tools in a single computer process:

- Kinect V2 (Microsoft, USA): This sensor implements depth and RGB images to segment the human body. In this work, the second version of this sensor was used with the Windows SDK, which provides 25 body points. It can measure the 3D position and orientation of each body point at a sample rate of 30 Hz. Moreover, because this activity is normally executed in the same plane, this Kinect has been widely used to analyze the STS movement, showing great accuracy and performance [73]. The sensor was placed on a tripod at 1 m from the floor and 4 m from the subject, as it is suggested for the right usage [73].
- Zephyr HxM BT (Medtronic, Ireland): This sensor is a wearable sensor that has been used to extract heart rate information of the patients requiring continuous monitoring. In this study, data were collected through a Bluetooth communication channel with a sample rate of 1 Hz. It was placed on the volunteer’s chest with an elastic band. Moreover, it is implemented to measure the resting heart rate of each subject. The selection of the Zephyr BT sensor was made based on accuracy, reliability, cost, availability, and comfort [74,75,76].
- Borg CR10: Aiming to simplify the explanation of this scale, Figure 1 was used to explain the meaning of the values to each volunteer, where it is possible to see the division of the fatigue levels (low, moderate and high). On the other hand, according to the results of the multi-dimensional fatigue inventory criteria, it was ensured that the participants were at a level of zero fatigue, i.e., non-fatigued condition. This scale was asked to the volunteer every 30 s during the STS test without interrupting the exercise. Hence, if the volunteer was able to complete the 2 min exercise, the corresponding test register ended up with 4 Borg CR10 values.

### 2.3. Procedure

Initially, each volunteer’s maximum heart rate (*MHR*) was estimated by implementing the “Tanaka equation”, shown in Equation (1). It uses the user age (*AGE*, in years) for getting an approximation of their *MHR*. It is essential to highlight that the Tanaka equation is recommended for healthy individuals such as those involved in this study because this equation significantly overpredicts maximal heart rate. Therefore, for people who present some diseases, it is recommended to adapt this method for exercise testing [77,78].

(1) MHR=206.9−(0.67∗AGE)

The volunteers were informed about the use of the Borg CR10 scale and instructed to warm-up for 5 min, composed of stretching movements and a 3 min treadmill walking. Afterward, the participants were instrumented with the Zephyr sensor. For the test, a 40 cm high chair without armrests was used, which was placed 4 m in front of the Kinect V2 sensor.

**Figure 2:**
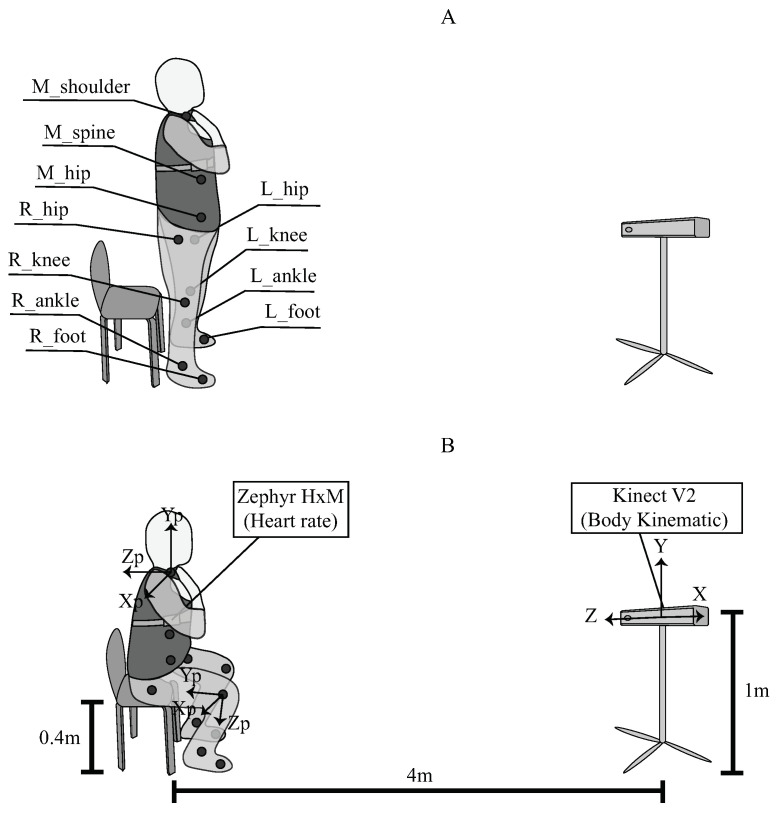
Set-up of the study and sit-to-stand representation, (**A**) standing position and (**B**) sitting position.

Before starting the test, participants were asked to stand with their hands on their shoulders and were instructed to look straight forward during the entire test. At the moment the participants heard the command “forward”, they began to perform the exercise. The exercise consisted of sitting down and getting up from the chair as fast as possible for 120 s (2 min) without stopping. Nevertheless, if the heart rate overcame 90% of the *MHR*, or a 10 Borg value was notified, the test was immediately concluded. Finally, the volunteers were instructed to perform a 5 min cool-down. The STS exercise representation and the study set-up can be seen in Figure 2.

Although the Kinect V2 provides 25 body markers, in Figure 2A, only the markers used for the data processing are shown. These correspond to 3 markers located in the middle part of the upper body right (M_[name of marker]), 4 in the right leg (R_[name of marker]), and 4 in the left leg (L_[name of marker]). Figure 2B illustrates the sitting position, the sensor locations, the reference system of the Kinect V2 (X, Y, and Z), and the orientation of some Kinect points (Xp, Yp, and Zp).

### 2.4. Data Processing

**Figure 3:**
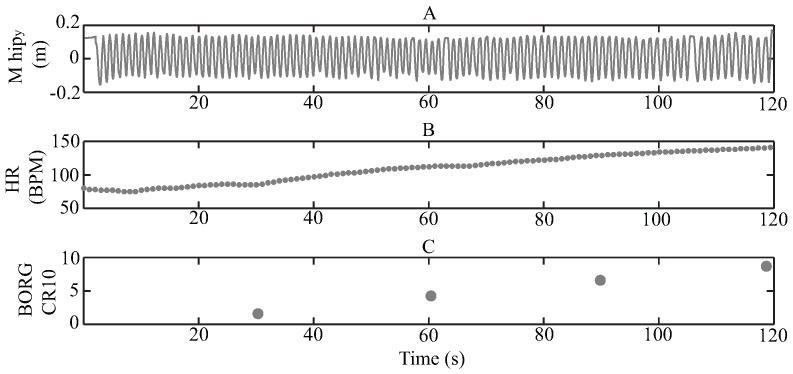
Example data of one volunteer test, (**A**) M_hip vertical signal, (**B**) heart rate signal and (**C**) Borg CR10 values.

Considering the metrics mentioned above, Figure 3 presents an example of a test register. In Figure 3A, the movement of the M_hip marker on the Y-axis ($M\_hip_{y}$) is shown, where it is possible to appreciate the sit-to-stand movement as a harmonic signal. This is because the STS test consisted of performing a repetitive activity, which creates a repetitive behavior in the position signals, especially for the vertical movements. Figure 3B illustrates the heart rate register and how it increments during the test. Finally, Figure 3C contains the 4 Borg CR 10 values mentioned by the volunteer every 30 s.

#### 2.4.1. Kinect Features

**Figure 4:**
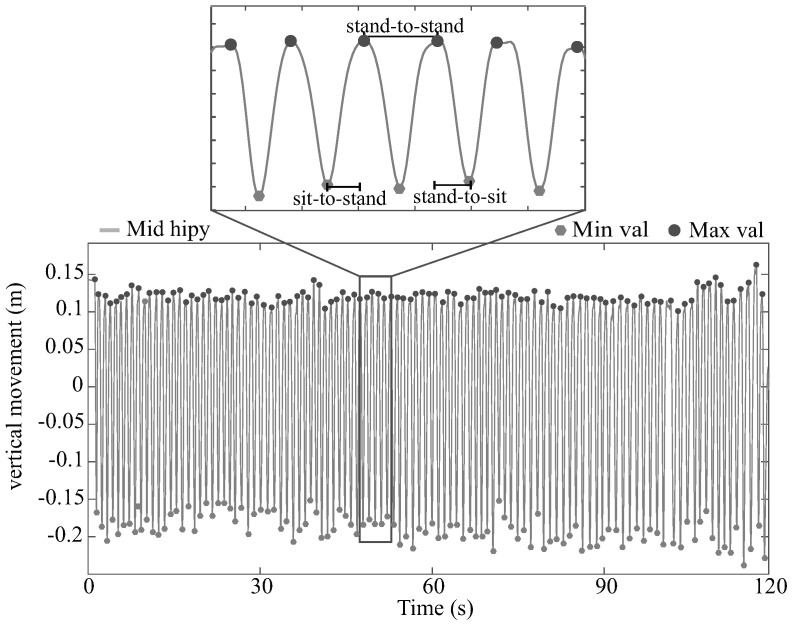
M_hip vertical movement signal, maximum, minimum and phase detection.

Taking advantage of the M_hip repetitive behavior on the Y-axis, an automated procedure was implemented to detect each stand-to-stand cycle. Essentially, the process consisted of subtracting the mean value of the whole M_hip signal and detecting the minimum and maximum values of each cycle. Therefore, these maximum values were considered as the moments when the volunteer was standing and the minimum values when the subject was sitting. Hence, these values allow estimating the two phases of the STS activity, stand-to-sit and sit-to-stand. Figure 4 presents an example of M_hip signal on the Y axis ($M\_hip_{y}$) of one test register, where it is possible to see the maximum values (Max_val) and minimum values (Min_val) of the corresponding signal. Furthermore, Figure 4 shows a zoom of one part of the signal, where a stand-to-stand cycle and its phases can be appreciated.

According to these stand-to-sit and sit-to-stand phases, the following 32 kinematic and temporal features were estimated for each stand-to-stand cycle, where “Fn” represented the feature number “n”, and the symbol “*” indicates that the corresponding feature was estimated with the mean value of both sides, left and right:

- F1: Stand-to-stand time (s), estimated with the duration of the stand-to-stand cycle.
- F2: Sit-to-stand time (s), estimated with the duration of the sit-to-stand phase.
- F3: Stand-to-sit time (s), estimated with the duration of the stand-to-sit phase.
- F4: M_hip vertical range (m), measured as the difference between the maximum and minimum value of the $M\_hip_{y}$ signal during the stand-to-stand cycle.
- F5: M_hip depth range (m), measured as the difference between the maximum and minimum value of the $M\_hip_{z}$ signal during the stand-to-stand cycle.
- F6: M_hip max vertical velocity (m/s), estimated by deriving the $M\_hip_{y}$ signal and obtaining its maximum value during the sit-to-stand phase.
- F7: M_hip min vertical velocity (m/s), estimated by deriving the $M\_hip_{y}$ signal and obtaining its minimum value during the stand-to-sit phase.
- F8: M_hip max depth velocity (m/s), estimated by deriving the $M\_hip_{z}$ signal and obtaining its maximum value during the stand-to-sit phase.
- F9: M_hip min depth velocity (m/s), estimated by deriving the $M\_hip_{z}$ signal and obtaining its maximum value during the sit-to-stand phase.
- F10*: Knee flexo-extension range (${}^{\circ }$). The knee flexo-extension signal was obtained by measuring the angle between the vectors composed by the hip, knee and the ankle Kinect 3D points (Figure 2A). Hence, this feature was estimated with the difference between the maximum and minimum value of the corresponding signal during the sit-to-stand phase (m/s).
- F11*: Knee flexo-extension max velocity (${}^{\circ }$), estimated by deriving the knee flexo-extension signal and obtaining its maximum value during the stand-to-sit phase.
- F12*: Knee flexo-extension min velocity (${}^{\circ }$), estimated by deriving the knee flexo-extension signal and obtaining its minimum value during the sit-to-stand phase.
- F13*: Hip flexo-extension range (${}^{\circ }$). The hip flexo-extension signal was obtained with the X-axis angle of the matrix rotation between the M_hip and the knee Kinect 3D orientation (Figure 2B). Hence, this feature was estimated with the difference between the maximum and minimum value of the corresponding signal during the sit-to-stand phase.
- F14*: Hip flexo-extension max velocity (${}^{\circ }$), estimated by deriving the hip flexo-extension signal and obtaining its maximum value during the stand-to-sit phase.
- F15*: Hip flexo-extension min velocity (${}^{\circ }$), estimated by deriving the hip flexo-extension signal and obtaining its minimum value during the sit-to-stand phase.
- F16*: Hip abduction-adduction range (${}^{\circ }$). The hip abduction-adduction signal was obtained with the Z-axis angle of the matrix rotation between the M_hip and the knee Kinect 3D orientation (Figure 2B). Hence, this feature was estimated with the difference between the maximum and minimum value of the corresponding signal during the sit-to-stand phase.
- F17*: Hip abduction-adduction max velocity (${}^{\circ }$/s), estimated by deriving the hip abduction-adduction signal and obtaining its maximum value during the stand-to-sit phase.
- F18*: Hip abduction-adduction min velocity (${}^{\circ }$/s), estimated by deriving the hip abduction-adduction signal and obtaining its minimum value during the sit-to-stand phase.
- F19*: Ankle flexo-extension range (${}^{\circ }$). The Ankle flexo-extension signal was obtained by measuring the angle between the vectors composed by the knee, ankle and foot Kinect 3D points (Figure 2A). Hence, this feature was estimated with the difference between the maximum and minimum value of the corresponding signal during the sit-to-stand phase.
- F20*: Ankle flexo-extension max velocity (${}^{\circ }$/s), estimated by deriving the ankle flexo-extension signal and obtaining its maximum value during the stand-to-sit phase.
- F21*: Ankle flexo-extension min velocity (${}^{\circ }$/s), estimated by deriving the ankle flexo-extension signal and obtaining its minimum value during the sit-to-stand phase.
- F22: M_shoulder vertical range (m), measured as the difference between the maximum and minimum value of the $M\_shoulder_{y}$ signal during the stand-to-stand cycle.
- F23: M_shoulder depth range (m), measured as the difference between the maximum and minimum value of the $M\_shoulder_{z}$ signal during the stand-to-stand cycle.
- F24: M_shoulder max vertical velocity (m/s), estimated by deriving the $M\_shoulder_{y}$ signal and obtaining its maximum value during the sit-to-stand phase.
- F25: M_shoulder min vertical velocity (m/s), estimated by deriving the $M\_shoulder_{y}$ signal and obtaining its minimum value during the stand-to-sit phase.
- F26: M_shoulder max depth velocity (m/s), estimated by deriving the $M\_shoulder_{z}$ signal and obtaining its maximum value during the sit-to-stand phase.
- F27: Spine flexo-extension range (${}^{\circ }$). The spine flexo-extension signal was obtained with the X-axis angle of the matrix rotation between the M_shoulder and the M_hip Kinect 3D orientation (Figure 2B). Hence, this feature was estimated with the difference between the maximum and minimum value of the corresponding signal during the sit-to-stand phase.
- F28: Spine flexo-extension max velocity (${}^{\circ }$/s), estimated by deriving the spine flexo-extension signal and obtaining its maximum value during the stand-to-sit phase.
- F29: Spine flexo-extension min velocity (${}^{\circ }$/s), estimated by deriving the spine flexo-extension signal and obtaining its minimum value during the sit-to-stand phase.
- F30: Spine abduction-adduction range (${}^{\circ }$/s). The spine abduction-adduction signal was obtained with the Z-axis angle of the matrix rotation between the M_shoulder and the M_hip Kinect 3D orientation (Figure 2B). Hence, this feature was estimated with the difference between the maximum and minimum value of the corresponding signal during the sit-to-stand phase.
- F31: Spine abduction-adduction max velocity (${}^{\circ }$/s), estimated by deriving the spine abduction-adduction signal and obtaining its maximum value during the stand-to-sit phase.
- F32: Spine abduction-adduction min velocity (${}^{\circ }$/s), estimated by deriving the spine abduction-adduction signal and obtaining its minimum value during the sit-to-stand phase.

**Figure 5:**
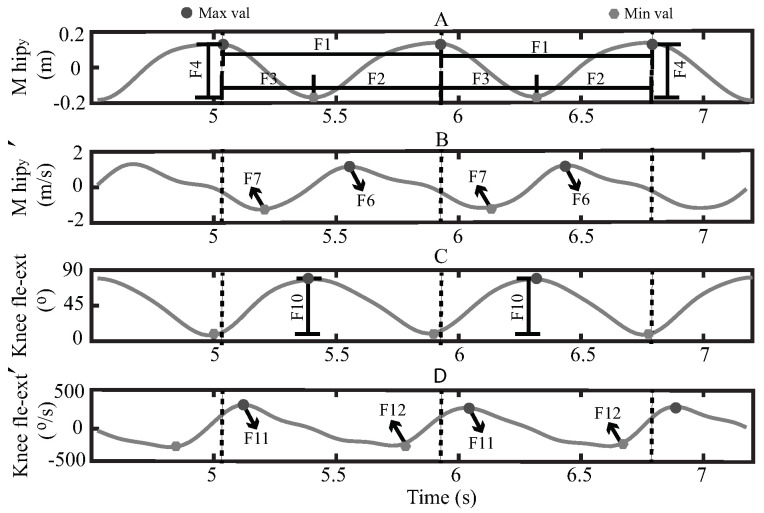
Features extraction process from the Kinect row data, (**A**) M_hip vertical movement signal, (**B**) M_hip vertical velocity signal, (**C**) nee flexo-extension signal and (**D**) Knee flexo-extension velocity signal.

Figure 5 illustrates an example of some different features estimation in two consecutive stand-to-stand cycles. The dashed lines contain the stand-to-stand cycles, the superscript symbol “ ’ ” represents the derivative operation of the corresponding signal, the dark dots presents the maximum values (Max_val) of each signal, and the gray polygons the minimum values (Min_val). Figure 5A presents graphically the estimation of the stand-to-stand time (F1), sit-to-stand time (F2), stand-to-sit time (F3) and M_hip vertical range (F4). Figure 5B shows the derivative of the $M\_hip_{y}$ signal ($M\_hip_{y}^{{}^{\prime }}$), the M_hip max and min vertical velocity (F6 and F7). Figure 5C shows the knee flexion-extension signal (Knee fle-ext) and the estimation of the Knee flexo-extension range (F10). Finally, Figure 5D presents the derivative of the Knee flexo-extension signal (Knee $fle-ext^{{}^{\prime }}$), the Knee flexo-extension max and min velocity (F11 and F12).

#### 2.4.2. Borg Interpolation, Features Relation and Heart Rate Incorporation

**Figure 6:**
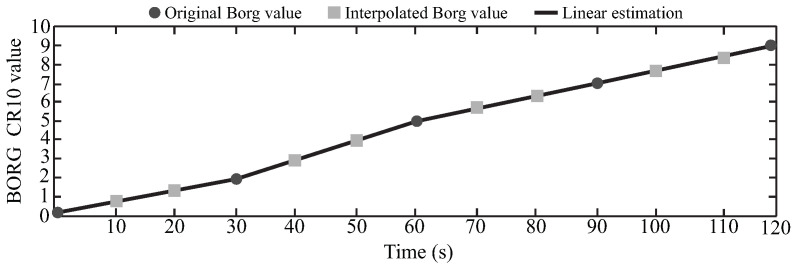
Linear interpolation of the Borg values every 10 s.

The 60 volunteers were able to finish the 2 min test; therefore, at the end of the data recollection, there were 240 Borg vales, 4 for each volunteer as it is illustrated in Figure 3C. However, only 8 subjects reported a 10 Borg CR10 value at the end of the test, which means that they reached the maximum fatigue level. Bearing in mind that the study aims to develop a computational model based on a data set and machine learning techniques, the Borg CR10 values were interpolated every 10 s employing linear estimation [79,80] to obtain more fatigue values. This estimation is based on the original 4 values and the assumption of the 0 Borg sate at the beginning of the test (according to the “multi-dimensional fatigue inventory” results). The idea consisted of estimating the 4 straight-line equations, by using the 4 provided Borg values and then calculating the Borg value at the corresponding time. Hence, after this process, every register contains 13 Borg values, considering 0 as the initial one. Figure 6 presents an example of this procedure, where the black dots represent the original Borg values, the gray squares the interpolated Borg values, and the black lines the lineal estimation.

**Figure 7:**
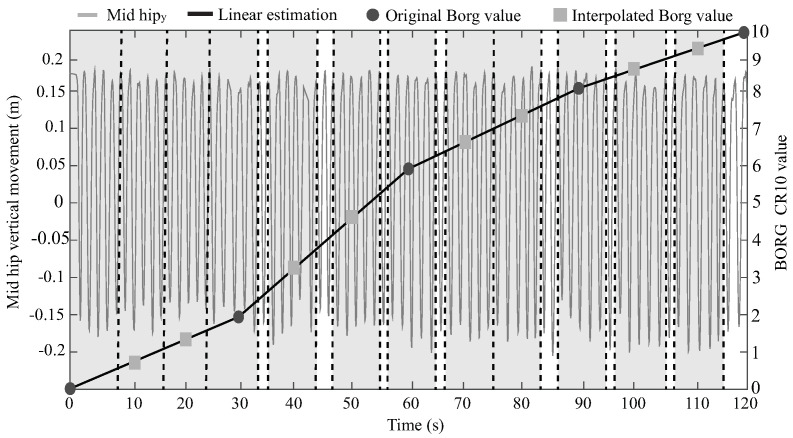
Selection of the five nearest sit-to-stand cycles to each Borg Value.

On the other hand, it is essential to highlight that the performance test is strongly dependent on each subject’s physical capability. Thus, the number of stand-to-stand cycles executed may be different, as well as the amount of STS features and their values. The lowest number of cycles obtained was 71, and the highest was 127. Consequently, to relate the fatigue level to each performance feature (F1 to F32), the five closest stand-to-stand cycles to each Borg value were used to estimate an average of each STS feature. This number of cycles was obtained by analyzing the ten registers with the least number of stand-to-stand cycles. Therefore, no cycle was repeated for the Borg values, except for the last one since the final part of the test is where the lowest cycle rate is presented, which does not allow to accomplish the non-repeated cycle requirements for all participants. Figure 7 illustrates an example of these nearest cycle selections, where the dashed lines with the gray light background contain the selected sit-to-stand cycles for each Borg value. Furthermore, it can be seen in the white background rectangles which cycles were not used and that the final Borg was not related to any cycle.

**Figure 8:**
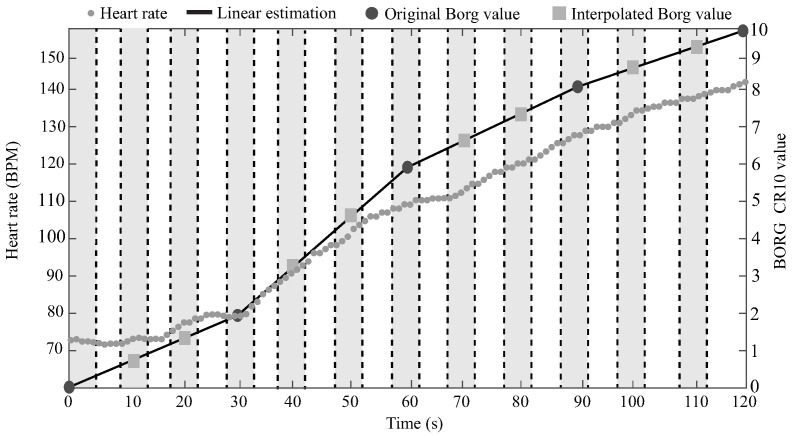
Selection of the five nearest heart rate records to each Borg value.

Considering the importance of the heart rate for the patient’s fatigue monitoring in the rehabilitation programs, this parameter was incorporated into the data set as the feature number 33 (F33) in a similar way as the other features. As the Zephyr sample rate is 1 Hz, each test register contains 120 heart rate records. Hence, aiming to get an average value without repeating records, the mean values of the five closest heart rate measurements to each Borg value (except for the last one) were used to relate the fatigue level with this physiological parameter. Figure 8 presents an example of these heart rate record selections, represented by the dashed lines and the clear gray background.

Therefore, at the end of this process, the interpolated and original Borg values are related to the average of the corresponding 32 kinematic/temporal features (F1 to F32) and the average heart rate (F33).

#### 2.4.3. Data Normalization

Feature variability caused by the subject physical condition makes it difficult to perform a direct comparison among the volunteer registers, which requires a normalization of the data according to each initial subject performance [81,82,83]. Hence, considering that all the volunteers were at a zero fatigue level at the beginning of the test and it is where the best performance should be presented, all features extracted were normalized by dividing it with the corresponding initial value (see Equation (2)).

(2) $$f_{i_{normalized}}=\frac{f_{i}}{f_{o}}$$

**Figure 9:**
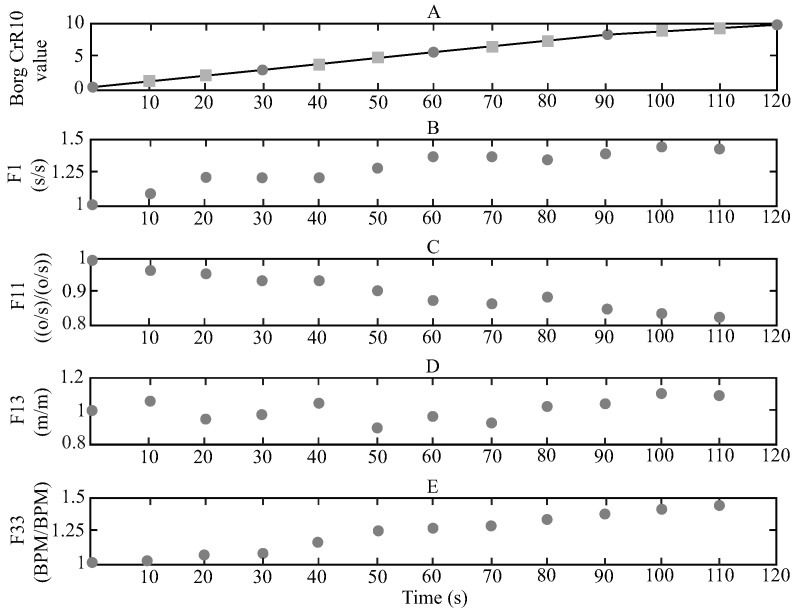
The behavior of the features normalized and fatigue level example, (**A**) Borg CR10 interpolated, (**B**) stand-to-stand time normalized, (**C**) Knee flexo-extension max velocity normalized, (**D**) Hip flexo-extension range normalized and (**E**) Heart rate normalized.

Figure 9 presents an example of one volunteer for three different features normalized. Figure 9A shows the Borg values reported and interpolated. Figure 9B exhibits the behavior of the sit-to-stand time (F1) and how this feature tends to increase. Figure 9C displays the behavior of the Knee flexo-extension max velocity (F11) and how this feature tends to decrease. Figure 9D shows the behavior of the Hip flexo-extension range (F13) and how this feature does not present a continuous increment or decrement. However, it illustrates constant behaviors in some parts of the test (like at the end of the test, where this feature results in higher values than the beginning). Finally, Figure 9E shows that the mean normalized values of the heart rate are increasing.

#### 2.4.4. Data Set Construction

After Borg interpolation and considering that the first Borg value was used to normalize the features, and the last Borg value was not used, 11 STS performance-related fatigue levels were finally obtained for each participant. Thus, at the end of the process, a total of 660 (60 participants × 11 fatigue levels) Borg registers related to the performance features were obtained for the data set.

**Figure 10:**
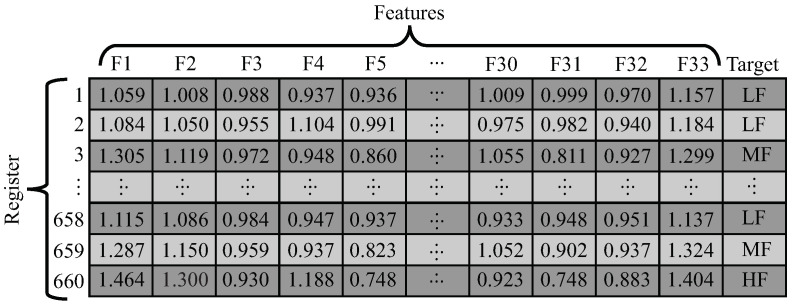
Data set representation composed of 660 STS registers, 33 features and the fatigue target.

To determinate the target for all registers, each one was labeled with 3 fatigue states (low, moderate and high, as illustrated in Figure 1) according to the corresponding Borg value. In such a way, registers with a related Borg value between 0 and 3 were considered as low fatigue (LF); between 4 and 6, as moderate fatigue (MF); and between 7 and 10, as high fatigue (HF). Thus, each register is composed of 33 normalized features (32 STS kinematic/temporal and 1 of the heart rate) and 1 target. The representation of the data set can be seen in Figure 10, where it can be seen how the 660 registers contain their corresponding features and targets.

Finally, to analyze in general how the features change according to the 3 fatigue categories, the mean and standard deviation were calculated for each feature regarding the fatigue condition. Therefore, it is possible to observe if the features, in general, present statistically different values and how those features behave concerning the fatigue.

#### 2.4.5. Fatigue Estimation Model

To develop and evaluate a computational model to estimate the three fatigue states using the 33 features, different machine learning methods were explored based on the obtained data set. Overall, the machine learning model development is divided into two phases: the training phase and the testing phase [84]. In the training phase, a huge part of the data set is used to train the model (usually, between 70% and 90%). Therefore, it can process the features and find patterns, regarding some training algorithms [84]. In the testing phase, the remaining part of the data set is used to assess the trained model by comparing the estimated outputs to the targets so that the model is evaluated with data that were not implemented for the training [84].

In this case, the training and test stage of the classifiers was conducted employing a specific technique called “cross-validation”. The classifiers model parameters were trained through leave-one-out cross-validation, which involves partitioning a sample of the size of “N” into a calibration sample of size “N-1” and a validation sample of size 1 and repeating the N process times. In this context, each model is trained with “N-1”. Different data groups were assessed with the reminder group [84]. Here, cross-validation is applied multiple times for different values of the tuning parameter, and the parameter that minimizes the cross-validated error is then used to build the final model. Thereby, cross-validation addresses the problem of overfitting [85]. In the end, this technique provides a general performance metric called “accuracy”, which is the relation between the total correct estimations obtained in each testing process or true positives (*TP*) and the complete amount of data (*N*), as it is shown in Equation (3).

(3) $$Accuracy=\frac{TP}{N}$$

**Figure 11:**
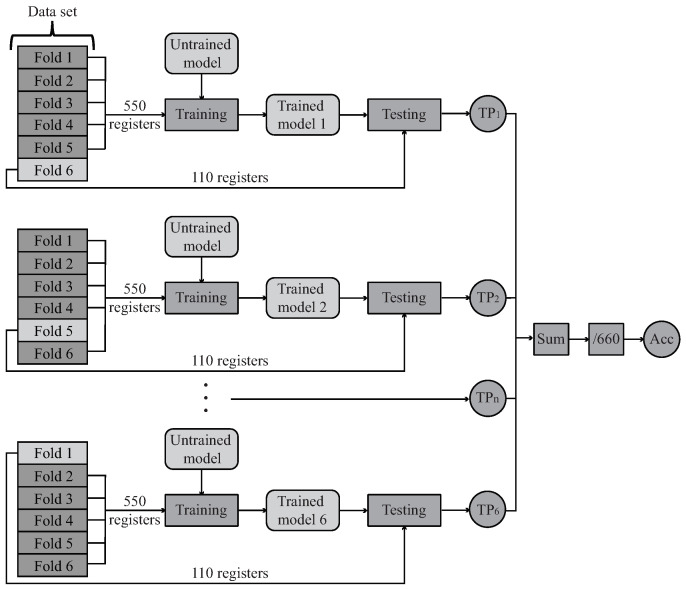
Cross validation process for the machine learning model training and assessment.

Considering the size of the data set, 6 folds were selected for this validation process; hence, each fold consists of 110 registers. Figure 11 illustrates this process, where “TPn” represents the number of true positives of the corresponding “n” iteration, and “Acc” represents the final accuracy metric.

Moreover, the false negatives (*FN*), that represent the amount register were estimated as other fatigue groups; and the false positives (*FP*), that refer to the number of registers that belong to other groups and were wrongly estimated, were calculated to obtain 3 more performance metrics known as “*Precision*” (Equation (4)), “*Recall*” (Equation (5)) and “*F-Score*” (Equation (6)).

(4) $$Precision=\frac{TP}{TP+FP}$$

(5) $$Recall=\frac{TP}{TP+FN}$$

(6) $$F1_{micro}score=\frac{Precision*Recall}{Precision+Recall}*2$$

From our framework’s perspective, it is impossible to predetermine which methods will work best for fatigue prediction because these methods are data-driven and, thus, are application-dependent, i.e., dependent on the exercise, extracted features, sensors, and scenarios, among others. Therefore, several methods were applied during our preliminary analysis of the data to develop the fatigue prediction model. The models evaluated included: logistic regression (LR), decision trees (DT), k-nearest neighbors (KNN), support vector machine (SVM), naive Bayes (NB), linear discriminant (LDA), artificial neuronal network (ANN), and random forest (RF). The open-source python library “scikit-learn” [86] was used to execute a quick general training for these classifiers. Afterward, according to the accuracy metric, and due to their relatively poor performance, DT, LDA, and NB were eliminated. Hence, our case study focused on using the best five classification models (LR, KNN, SVM, RF, ANN), adjusted and retrained, by modifying their training parameters automatically through computational iterations. The theoretical approach of the machine learning models used is summarized below.

A statistical model such as LR attempts to build a relationship among the input variables and response employing parametric methods. In other words, it uses a logistic function to model conditional probability. Hence, LR is a supervised learning algorithm technique where the probability of a dichotomous outcome is a function of the predictors/features [87,88]. Although LR is a simple yet very effective classification algorithm, its performance can vary significantly with sparse data [88]. Moreover, non-parametric approaches such as KNN, SVM, and ANN, are commonly used in human performance modeling applications [89,90,91,92]. KNN is a simple classifier, an easy-to-implement supervised machine learning algorithm that can solve both classification and regression problems. The algorithm assumes that similar things are near to each other; therefore, it requires the computation of the distance of the unlabeled object to all the labeled objects in the training set [93]. Regarding the SVM classifier, which is a supervised learning method that uses kernel functions for data classification and regression analysis, its methodology consists of using a hyperplane to separate one-dimensional data to a high-dimensional space from a given labeled data set [94,95] to identify the optimal hyperplane to classify the given data with minimum error [95].

Concerning the ANN classifier, it is a supervised machine learning classifier that seeks to classify an observation as belonging to some discrete class based on inputs. This classifier is a set of connected input-output networks in which weight is associated with each connection. It consists of one input layer, one or more intermediate layers, and one output layer. Learning of neural networks is performed by adjusting the weight of the connection. By updating the weight iteratively, the performance of the network is improved [96]. Finally, concerning the RF model, the ensemble classification algorithm utilizes trees as base classifiers to generate many classifiers and aggregate their results via voting. It means that each tree in the random forest spits out a class prediction and the class with the most votes becomes our model’s prediction. The premise of this method is that combining a large number of single classifiers allows for a more diverse representation of the data and consequently a more accurate prediction [97,98,99].

## 3. Results

Table 2 shows the descriptive data of the number of stand-to-stand cycles obtained in the 60 registers, specifically, the mean, median, standard deviation, maximum, and minimum cycle number. It is possible to see that, on average, the subjects executed 97.24 stand-to-stand cycles, which means that in general, the cycle rate was 0.803 cycles per second. Furthermore, it shows in the table that the minimum stand-to-stand cycle number achieved was 71, and the maximum was 127, obtaining a difference of 56 cycles.

Table 3 presents the number of registers for the three fatigue states, according to the labeling process presented at the end of Section 2.4.4: low fatigue (LF), moderate fatigue (MF) and high fatigue (HF). It can be seen that the MF group contains most of the registers, followed by the LF group. Hence, the HF group has the lowest value, showing a difference of 57 registers regarding the MF group, which corresponds to 8.6% of the total data.

**Figure 12:**
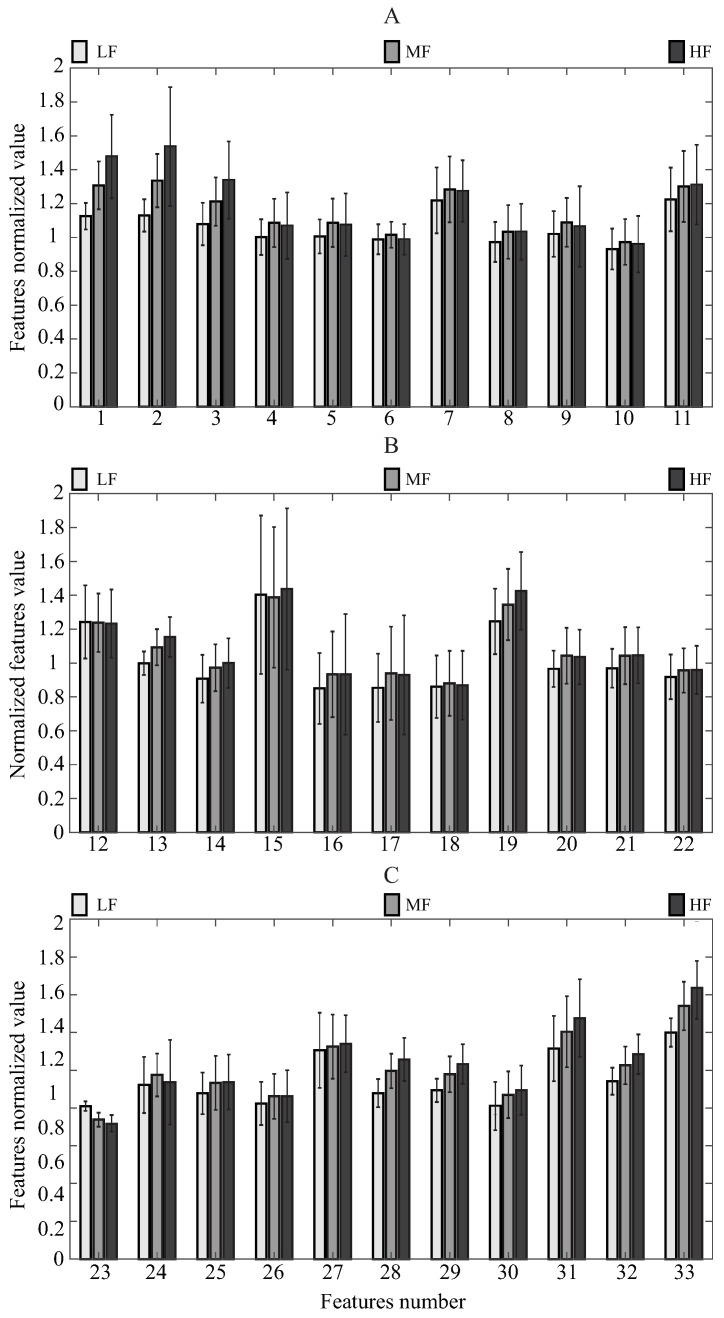
Mean and standard deviation values of each feature, according to the 3 fatigue conditions. (**A**) corresponds to the features values between 1 to 11; (**B**) corresponds to the features values among 12 to 22; (**C**) corresponds to the features values between 23 to 33.

Figure 12 displays the mean (bars) and standard deviation (black lines) of each normalized feature, regarding the fatigue state, where the light gray bars correspond to LF, the gray bars to the MF, and the black bars to the HF. Furthermore, the features are split into 3 different bar graphs; hence, Figure 12A contains features from 1 to 11, Figure 12B from 12 to 22, and Figure 12C from 23 to 33. It is essential to highlight that these features are almost close to 1, owing to the normalization process carried out in Section 2.4.3, which allows comparing the feature behaviors among them. Therefore, it can be appreciated how some features increment or decrement their statistical values according to the fatigue condition, presenting that, in general, their values have changed. Among the features, the stand-to-stand time (F1), the sit-to-stand time (F2), the heart rate (F33), Spine flexo-extension max velocity (F28), and M_shoulder depth range (F23) show the highest changes. Regarding the LF, MF and HF: F1 has mean values of 1.125, 1.307 and 1.477; F2 values of 1.130, 1.335 and 1.537; F33 values of 1.340, 1.497 and 1.605; F23 values of 0.904, 0.824 and 0.799; and F28 values of 0.981, 1.113 and 1.181. Finally, there are features that do not illustrate high changes. These features correspond to the M_hip min vertical velocity (F7), M_hip max depth velocity (F8) and hip abduction-adduction max velocity (F17).

**Figure 13:**
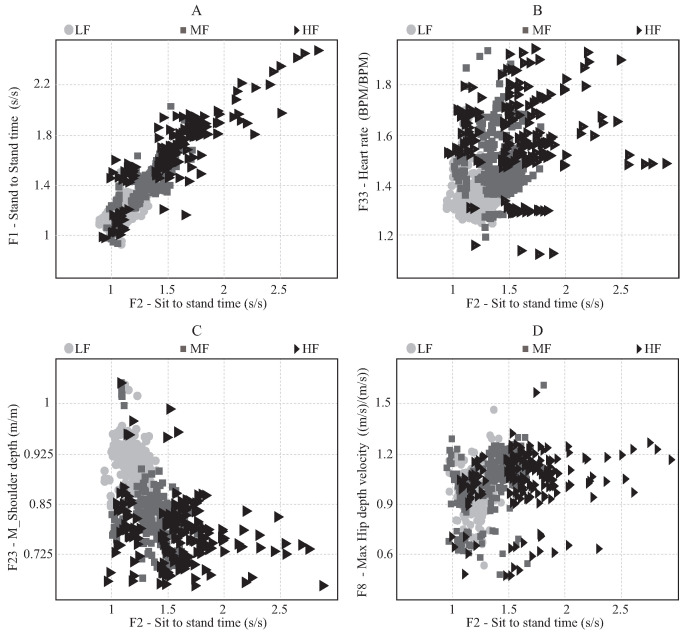
Features scatter graphs regarding the stand-to-stand time, (**A**) stand-to-stand time vs. sit-to-stand time, (**B**) sit-to-stand time vs. heart rate, (**C**) sit-to-stand time range vs. M_shoulder depth range, and (**D**) sit-to-stand time vs. M_hip max depth velocity.

Figure 13 presents four examples of the data distribution regarding two specific features, using the sit-to-stand time (F2) always as the horizontal axis. Therefore, the black triangles represent the high fatigue registers; the gray squares, the moderate fatigue; and the clear gray circles, the low fatigue. Specifically, Figure 13A shows the distribution according stand-to-stand time (F1); Figure 13B, according to the heart rate (F33); Figure 13C, according to the M_shoulder depth range (F23); and Figure 13D, according to the M_hip max depth velocity (F8). Essentially, these plots display some patterns that can be found in the data set, where it is possible to see how some features are related (Figure 13A) and others not (Figure 13D). Considering the number of features, there are 33 scatter plot options; hence, in Figure 13, only the most relevant features are shown, which were chosen considering Figure 12.

**Figure 14:**
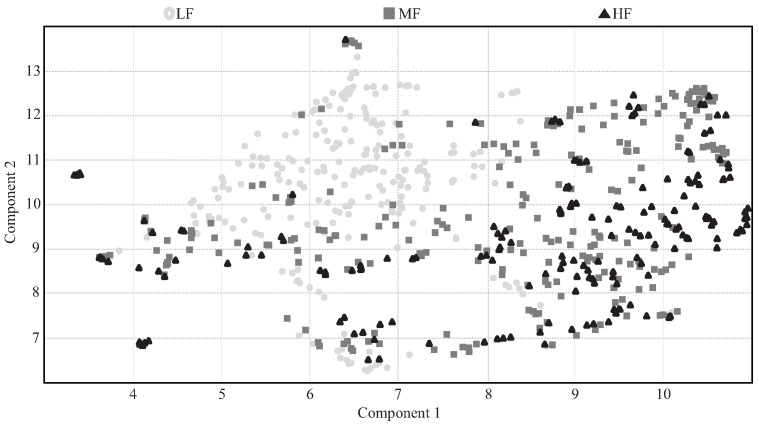
Distribution diagram obtained from applying the Uniform Manifold Approximation and Projection technique.

The Uniform Manifold Approximation and Projection (UMAP) was implemented to provide a 2D representation of how the data are distributed among the three classes. The UMAP algorithm allows us to represent the features into a reduced number of components. These components are used for visualizing and estimating possible clusters among the classes [100]. Hence, Figure 14 presents the obtained 2D representation of reducing the 33 features into two components, where the LF registers are easily separated from the MF and HF registers. However, the UMAP technique does not display a clear separation between the MF and HF registers.

**Figure 15:**
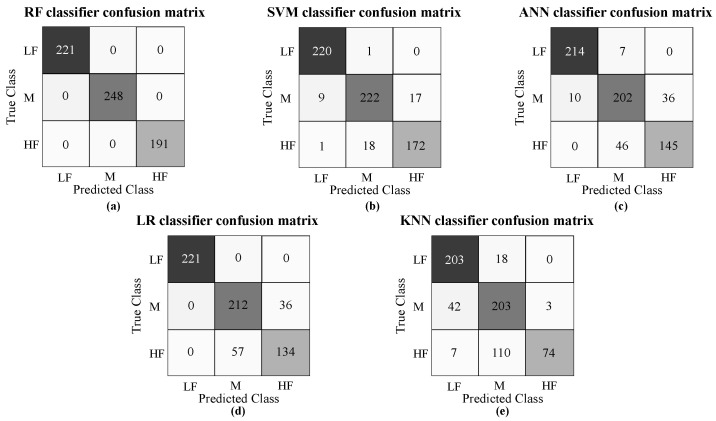
Confusion matrix of (**a**) RF classifier, (**b**) SVM classifier, (**c**) ANN classifier, (**d**) LR classifier, and (**e**) KNN classifier.

The confusion matrix obtained from the best five classifier models implemented after exploring in a grid search manner with the data obtained in Section 2.4.5 is shown in Figure 15, where along the x-axis are listed the true class labels and along the y-axis are the class predictions. Along the first diagonal are the correct classifications, whereas all the other entries show misclassifications. In the same way, Table 4 reports the parameters and the performance of the five classifiers implemented. The k-nearest neighbor (KNN) method using the Euclidean distance classified the registers by a majority vote of its nearest elements with 12 neighbors (K = 12). The logistic regression (LN) classifier implements the large-scale bound-constrained optimization as a penalty algorithm (solver = lbfgs) and a value of 1000 for its inverse of regularization strength learning parameter (C = 1000). Then, it implements the artificial neuronal network with a stochastic gradient-based optimizer (solver=adam), and 100, 20, and 100 as hidden layer sizes (hls = (100, 20, 100). The support vector machine (SVM) has a radial basis function kernel (kernel = rbf) and a constrain value of 2 (C = 2). Finally, the best model is a random forest classifier with 60 estimators (n_estimators = 60), which means that the model integrates 60 decision tree models to merge their prediction. Moreover, Table 4 provides the mean values of the performance metrics: accuracy, recall, precision, and F-score, where the random forest (RF) model presents the highest values.

**Figure 16:**
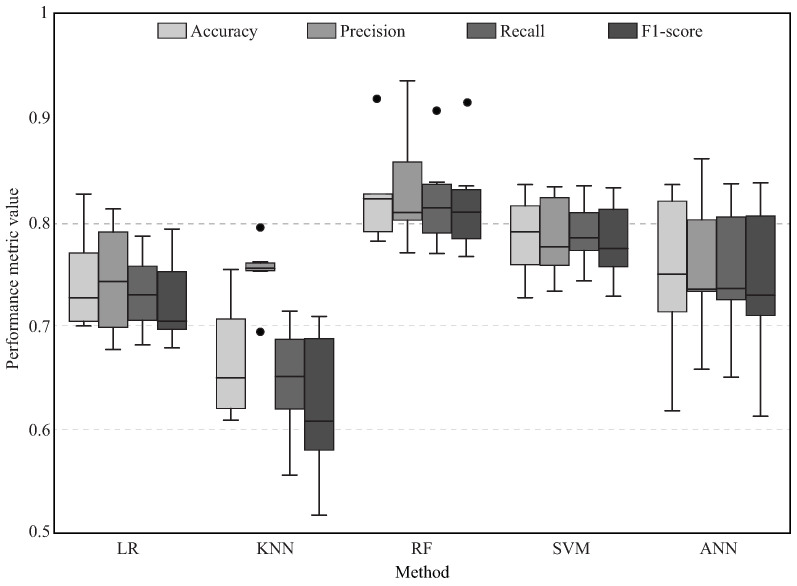
Box plot of the performance metric results for the five best machine learning methods.

Taking into account that the values in Table 4 are the mean values obtained after the six tests of the cross-validation process (Figure 11), Figure 16 presents the box plot of each reliability metric for the five machine learning implemented methods. Hence, each method contains four box plots, where the middle horizontal line represents the median value, the four quartiles are contained by the vertical lines, and the boxes and the black dots are atypical data. It can be seen that the RF method always presents the highest values, showing the lowest dispersion and, therefore, the lowest variance.

**Figure 17:**
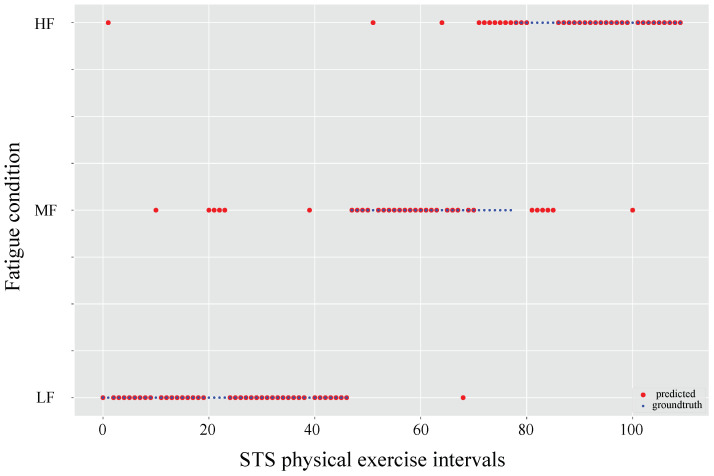
STS physical exercise intervals vs. fatigue conditions on the test set. The thinnest dots represent the ground-truth values, and the thickest dots are fatigue predictions.

Considering the above results in Figure 17, the data was sorted to systematically evaluate the performance of the random forest classifier in the fatigue condition prediction task. In addition, the classifier reported an outstanding response without showing problems related to overfitting or underfitting.

**Figure 18:**
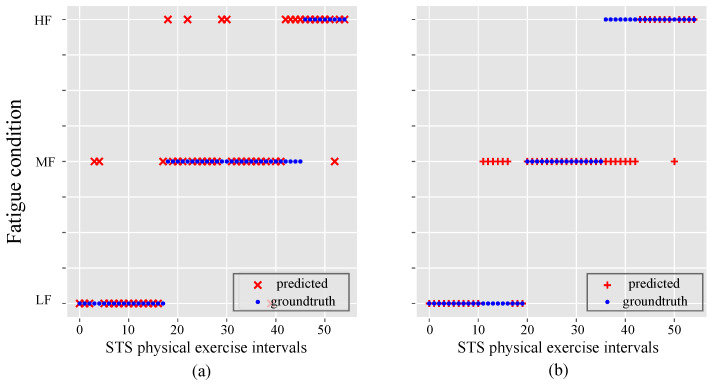
Gender comparison of STS physical exercise intervals versus fatigue condition on the test set. Crosses and × symbols describe the ground-truth values, and the thickest dots present; (**a**) represents the fatigue condition in STS physical exercise intervals in male patients and (**b**) represents the fatigue condition in STS physical exercise intervals in female patients.

To observe whether there is any gender effect on the RF classifier performance. The data were split to analyze the effect of gender on the fatigue condition prediction task individually, as reported in Figure 18. The two individual RF classifiers were trained separately for the male (a) and female (b) genders and showed high agreement between the fatigue condition and RF predictions.

**Figure 19:**
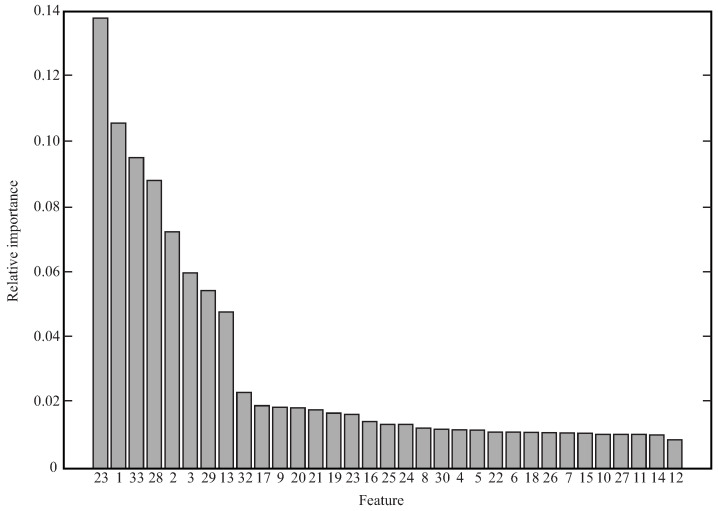
Relative importance of Features for the random forest model.

Finally, the feature importance property of random forest was explored to quantify the importance of each feature for the corresponding model. This property measures a relative weight value to each feature, which represents a direct relation to the importance of the corresponding feature for this classic machine learning model. Figure 19 presents as a bar graph, the relative importance values obtained for each feature, sorted from the highest to the lowest values. Then, F23 (M_shoulder depth range), F1 (stand-to-stand time), and F33 (heart rate) features present the highest values for our experimental set-up.

## 4. Discussion

According to previous works [101] that have studied the reference number of cycles in a 1 min STS test for healthy people, the results obtained in Table 2are lower. Specifically, the authors in [101] reported that subjects between 20 and 24 years have an average stand-to-stand rate of 1.183. Whereas in our results reported in Table 2, it is illustrated that the participants presented an average of 0.803. This might be attributed to the fact that the STS test performed by the participants in this work was twice as long (i.e., 2 min), which makes the test harder, and hence, the general performance decreases when they start to feel exhausted. Therefore, these results suggest that people decrease their performance when they begin to feel fatigued, i.e., their speed/intensity in performing the test decreases.

Similarly, this behavior was observed in all participants (regardless of their physical condition), where the rate of the cycle was not constant, and it tended to decrease during the test due to induced fatigue. This means that at the end of the test the number of cycles decreases. Therefore, regardless of whether the lowest number of cycles was executed (71) or the highest (127), the behavior was the same. Hence, five cycles were used to get the average for each feature for the data set and to ensure homogeneity in the data.

Although every volunteer started in a low fatigue condition, results in Table 3 display that most of the registers belong to moderate fatigue (MF). In contrast, the lowest register number is presented for the high fatigue group (HF). Considering that reaching a Borg value in the HF band requires to pass firstly for the LF and MF groups, this result was expected. However, a difference of 8.6% (57 registers) is acceptable for data analysis and training computational models [84]. Moreover, these results present that the data set registers are distributed similarly among the three fatigue groups. In general, the volunteers experimented with the three fatigue states during the test.

Bear in mind, previous studies have demonstrated that the times of the sit-to-stand phases are the most relevant exercise performance features [40]. The results in Figure 12 are concordant with the literature. An increment of approximately 20% can be observed for these types of features (F1 and F2) between the mean values of the LF and HF groups. However, despite the fact that the use of heart rate is criticized for managing the patient’s fatigue condition during HIE [50]. The results reported a direct relation with fatigue level. The heart rate (F33) has a difference of 21.7% between the LF and HF groups. Thus, the heart rate provides relevant information related to the individual’s fatigue state.

Nonetheless, other features present the opposite behavior. Specifically, the M_hip depth range (F5), the knee flexo-extension max velocity (F11), knee flexo-extension min velocity (F12), Hip flexo-extension min velocity (F15), M_shoulder vertical (F22) range, and M_shoulder depth range (F23) present the highest decrements. Because these features are related to the phase’s time and the movement of the spine, it is normal that the lower limb angular velocities decrease, especially the minimum velocities that correspond to the sit-to-stand phase.

However, features that come from the upper part of the body, specifically the M_shoulder movement ranges (F22 and F23), also decrease. This behavior may be due to the fact that the volunteers tried to change the exercise execution technique, to continue the activity as fast as possible, and to relieve the load on the main lower limb muscles. Furthermore, by moving the chest and the back to the front part, the exercise becomes easier [102], which causes the upper movement range features to decrease. Thus, although these features do not change as much as the time phase features and the heart rate, they also provide important information about the fatigue condition. It also contributes information about any possible variation in the correct execution of the exercise, which is essential for avoiding injury.

The relationship between the features themselves and the fatigue condition can be better analyzed in the 2D plots of Figure 13. In Figure 13A, the data distribution follows a straight line due to the fact that the sit-to-stand time is part of the stand-to-stand time; hence, both features are very related. It can be seen that the HF samples are clustered around the highest values. In contrast, the LF samples tend to be grouped in the lowest values. However, this graph does not present a clear difference between the LF and MF samples. Moreover, some irregular HF registers are in the lowest values, which makes it difficult to differentiate them from the other fatigue categories just with these two features, showing that just one parameter is insufficient for a good classification.

Figure 13B exhibits the data distribution regarding two features that are not related, the sit-to-stand time and heart rate. Hence, the samples are more dispersed and do not follow a precise equation. As above, the LF samples tend to be grouped in the lowest values; however, it can be seen that some LF registers reach values of about 1.6. This means that during the test, the heart rate reached values of approximately 60% higher than the repose heart rate of the corresponding volunteer and do not overcome a value of 1.5 of the sit-to-stand time. This represents exercise conditions where the volunteers were requiring more energy for doing the exercise and did not feel fatigued, and thus, they were able to keep a similar performance. Taking into account that these heart rate values are acceptable in some rehabilitation scenarios (e.g., oncology rehabilitation), this case may be optimal for physical training [3,12]. Nevertheless, by monitoring just the heart rate, it would be difficult to distinguish this optimal training condition from the cases where moderate or high fatigue levels are reached.

On the other hand, it is possible to see HF samples that do not overcome a 1.4 value in the heart rate and, they are in the highest values of the sit-to-stand time. These registers represent cases when the cardiac system was not able to adapt as fast as the exercise requires, which might happen in high-intensity exercises and are very dependent on the subject’s cardiorespiratory capability [50], and hence, they felt exhausted and were not able to keep executing the exercise with similar performance. However, it is possible to see the opposite case, where some HF and MF samples are presented in the heart rate highest values and in the lowest values of the sit-to-stand time. This case shows conditions where the volunteers felt compelled to adapt their execution technique to keep performing the activity quickly. Thus, it is important to monitor other exercise performance features where these changes can be appreciated.

The execution exercise technique change and its influence on the sit-to-stand time can be appreciated in Figure 13C, where many HF samples are grouped in the lowest values of the M_shoulder depth range. Considering that moving the back to the front facilitates the execution of the exercise and reduces the upper body displacement on the Z-axis [102], the exercise phase times tend to decrease, showing a better performance. However, the real situation reflects a pattern that, owing to the fatigue condition, the volunteers may modify their posture to reduce the load on the lower limbs. Therefore, the LF samples are clustered in the highest values of the M_shoulder depth range. In Figure 13B,C, it is possible to see how the fatigue distribution changes in both axes. In contrast, Figure 13D presents the data distribution of the M_hip max depth velocity, which does not provide a clear pattern visually. Thus, it is not possible to determinate data groups, clustered on the vertical axis, despite the fact that the feature changed its values in a similar range of the sit-to-stand time.

In addition, Figure 14 presents in general that the LF registries are easier to classify because they tend to be clustered according to the UMAP features reduction technique. Although Figure 14 does not present a clear separation between the MF and HF groups, this can be appreciated better in Figure 13 where the data tend to be clustered in specific ranges of the features. Specifically, it is possible to see that in the extreme values, the HF registers are normally shown.

Regarding the different patterns that can be presented and the number of features, one of the best ways to analyze the data set is by employing computational models capable of determining these and other behaviors. It can be seen in Figure 15 and Figure 16 and Table 4 that the machine learning model with the lowest reliability values is the KNN, which is based on distance techniques for classifying. Hence, considering the data distribution presented in the scatter plots (Figure 13 and Figure 14), it is possible to see that this is not the recommended method for this type of data. Despite the fact that the SVM and the ANN present a better performance estimation, these models based on estimating curves for classifying do not present the best performance because the groups are not quite separated. Hence, the RF model has the best reliability results. Considering the different cases that may be presented, this result suggests that the best method consists of merging different estimators that analyze the entire data to provide a consensual result.

We considered the common problems related to overfitting or underfitting in classifiers and also the consequence of these incorrect results, i.e., these can be divided into false positives or false errors. This is not desirable in clinical scenarios given the problems involved (overtraining, injury, affecting the patient’s rehabilitation process, among others). Therefore, the performance of the RF classifier in the fatigue state prediction task was evaluated, as illustrated in Figure 4. This result reported an outstanding classifier response without showing problems related to underestimation or overestimation of fatigue.

On the other hand, the RF classifier performance was analyzed as a function of the participant’s gender (male or female), as shown in Figure 18. The results showed a high agreement among fatigue conditions and RF predictions, i.e., the classifier performance does not decrease, and apparently, this consideration could improve RF performance. However, to ensure this hypothesis, it is necessary to test, the classifier on at least 100 patients. Therefore, the above is considered as future work, where we contemplate analyzing the relationship between gender with the classifier performance and their fatigue condition.

We regarded the feature importance obtained for each variable to indicate which features from the data are the most relevant in the training of a random forest model, as illustrated in Figure 19. Our proposed model describes the most important parameters related to the fatigue condition in STS exercises, such as M_shoulder depth range (F23), stand-to-stand time (F1), and heart rate (F33). These results are concordant to the STS study by Jimenez et al. [42], which reported that the acceleration of the chest is strongly related to the fatigue condition, considering that people try to move their upper body part to make the STS execution easier [102]. Moreover, Aguirre et al. [65] reported that considering that the stand-to-stand time contains information about both STS phases, it is the one that presents the most effective linear relationship according to the fatigue level. In the same way, Figure 19 shows the characteristics that do not provide any relevant information about the individual’s fatigue state in the execution of the STS exercise. These features correspond to those representing movements in the frontal plane, such as the abduction-adduction movement. This is because the STS exercise is performed primarily in the sagittal plane; therefore, these characteristics do not change completely or change randomly. This information may suggest a better understanding to clinicians of the parameters that should be analyzed to monitor the patient fatigue state in STS exercise with a limited number of parameters.

On the other hand, regarding the performance of machine learning models, it would be possible to perform a better feature selection, which could equalize or even increase the performance metrics of the classifiers by removing unnecessary features from the data. In future real-time applications, the dimensionality of data is vital to optimizing computational costs and running time.

Comparing to other similar studies [42,65], to the authors’ knowledge, this work is the first that presents a model for fatigue estimation with three states (low, medium, high) during the STS exercise execution by monitoring kinematic/temporal features and the heart rate. Regarding the study by Jimenez et al. [42], the authors demonstrated that chest acceleration in vertical motion is related to fatigue, using an accessible and practical device, the IMU of a smartphone. However, it presents one case study and only analyzes one kinematic feature that may change its behavior if the subject modifies the execution technique. On the other hand, in the research by Aguirre et al. [65], the authors carried out an analysis methodology to determine which STS features are significantly linearly related to the fatigue level, measured with the Borg CR10 scale. However, it only presents a linear analysis and does not analyze the different patterns and behaviors that can be presented.

Similar studies that proposed fatigue estimation models during different exercises or activities employing IMUs, such as walking [70], vertical jumps [69], lower limb endurance [66], frameworks activities related to manufacturing tasks [99], have shown accuracy values between 85% and 95%. Therefore, contrasting the proposed ensemble model performance with the literature, its results are in the lowest part of the range (83.2%). However, it must be considered that these similar studies only considered two fatigue conditions, fatigued and no-fatigued. In contrast, this work contemplates three states, increasing the probability of failing in the estimation but providing a clear separation for LF records with respect to MF and HF. (Figure 14). Moreover, this model allows more concrete monitoring of the individual’s fatigue level during the rehabilitation process, and with it, the possibility to improve the individual’s performance during therapy.

Even though the proposed model is not based on IMUs, it implements a KinectV2 for obtaining the exercise features, which is an affordable sensor that has shown to be helpful in clinical scenarios and allows to measure more STS features [73]. Considering the different patterns that may be presented in the lower and upper body parts, this sensor exhibits several advantages at being able to extract relevant STS features from different body parts. This allows to have constant monitoring of the person’s exercise execution technique and, thus, avoid any kind of injury. Furthermore, owing to the relevant heart rate information regarding the fatigue condition and its facility for being measured, this model also integrates an affordable heart rate sensor.

One limitation of this work is related to the study population because all the volunteers were healthy people, and the features may show different behaviors and patterns with patients or other groups with different physical conditions. However, the normalization process means that the model compares the user’s state with his/her initial condition, reducing the difference features variability presented among the volunteers. Furthermore, other similar studies also recruited healthy subjects [42,65,66,69,70]; hence, as a first approximation for a complete clinical tool, this work presents relevant results.

Finally, owing to the global confinement caused by COVID19, the need for clinical tools for telemedicine has significantly increased [71]. Hence, keeping in mind the importance of fatigue monitoring in physical rehabilitation and the practical tools that it implements, this work presents the initial development of a potential clinical tool for estimating fatigue during one of the most implemented HIE in rehabilitation programs.

## 5. Conclusions

First of all, a study was carried out to obtain a data set of 660 sit-to-stand registers. It was composed of 32 kinematic/temporal exercise features and the heart rate, each characteristic labeled with a fatigue condition (low, moderate, and high) based on the Borg scale values provided by the participants.

An analysis process was carried out to determine the most relevant features related to the fatigue condition. For this purpose, the behavior and pattern of each extracted characteristic were analyzed. Results suggest that the most important feature is the depth displacement of the upper body part, followed by the stand-to-stand time and the heart rate. Therefore, it is possible to suggest that the user’s physiological condition, the upper body features, and the lower body features contain relevant information regarding fatigue estimation during the STS exercise.

Finally, an approach of a fatigue estimation model is proposed aiming to show that these features can be implemented for estimating fatigue with an accuracy of 82.5% with accessible and practical sensors, which, according to similar studies, is in the acceptable range. Furthermore, this model allows classifying three fatigue conditions: low, moderate, and high. This allows for improved monitoring of the individuals’ fatigue state, thereby optimizing their performance and, consequently, the execution of the exercises. Hence, this work presents the development of a potential tool for physical rehabilitation scenarios and telemedicine applications that has become an important area during this global emergency caused by COVID19. 

## Figures and Tables

**Table 1:** Volunteer descriptive data (M ± SD).

Gender	Age (Years)	Weight (kg)	Height (cm)
Female	20.8±1.7	59.3±5.5	164.1±7.7
Male	21.9±1.9	65.9±6.4	172.8±8.3

**Table 2:** Descriptive data of the number of stand-to-stand cycles.

Mean	Median	SD	Maximum	Minimum
97.24	95	18.60	127	71

**Table 3:** The number of registers for each fatigue state.

Fatigue State	Number of Registers
LF	221 (33.5%)
MF	248 (37.6%)
HF	191 (28.9%)

**Table 4:** Performance of the five best fatigue estimation models. Bold values show the best score for each performance metric.

Model	Main Parameters	Overall Accuracy (%)	Precision (%)	Recall (%)	F-Score (%)
**RF**	n_estimators = 60	**83.2%**	**83.6%**	**83.0%**	**82.7%**
SVM	kernel = rbf class_weight =’balanced’	78.6%	78.5%	78.9%	78.1%
ANN	activation = ’relu’ solver = adam hls = (100,20,100) alpha = 0.05 learning_rate = ’adaptative’ max_iter = 1000	76.0%	77.1%	74.8%	75.0%
LR	solver = lbfgs C = 1000	74.4%	74.4%	73.2%	72.4%
KNN	k = 12 n_neighbors = 27	66.6%	75.2%	64.7%	62.1%

## Data Availability

Publicly available datasets were analyzed in this study. This data can be found here: https://figshare.com/articles/dataset/STS_fatigue_data_zip/15001362.

## Abbreviations

The following abbreviations are used in this manuscript:

PE	physical exercise
HRPF	Health-related physical fitness
$VO2_{MAX}$	maximum oxygen volume
LIE	light-intensity exercise
MIE	moderate-intensity exercise
HIE	high-intensity exercise
STS	sit-to-stand
MET	metabolic equivalent
VO${}_{2}$	oxygen uptake
HR	heart rate
HRR	heart rate reserve
EMG	electromyography
COVID19	coronavirus disease 2019
M	mean
SD	standard deviation
MHR	maximum heart rate
M_hip	middle hip Kinect marker
M_spine	middle spine Kinect marker
M_shoulder	middle shoulder Kinect marker
Max_val	maximum value
Min_val	minimum value
LF	low fatigue
MF	moderate fatigue
HF	high fatigue
TP	True posotive
FN	False negatives
FP	False positives
LR	Linear regression
RF	random forest
ANN	artificial neuronal network
SVM	support vector machine
KNN	k-nearest neighbor
UMAP	uniform manifold approximation and projection

## References

[1] Thompson P. (2003). Exercise and Physical Activity in the Prevention and Treatment of Atherosclerotic Cardiovascular Disease: A Statement From the Council on Clinical Cardiology. Arterioscler. Thromb. Vasc. Biol..

[2] World Health Organization (2014). Global Status Report on Noncommunicable Diseases 2014.

[3] Warburton D.E.R., Nicol C.W., Bredin S.S.D. (2006). Prescribing exercise as preventive therapy. CMAJ.

[4] Pedersen B.K. (2019). Physical Exercise in Chronic Diseases. Nutrition and Skeletal Muscle.

[5] Ignarro L.J., Balestrieri M.L., Napoli C. (2007). Nutrition, physical activity, and cardiovascular disease: An update. Cardiovasc. Res..

[6] Price K.J., Gordon B.A., Bird S.R., Benson A.C. (2016). A review of guidelines for cardiac rehabilitation exercise programmes: Is there an international consensus?. Eur. J. Prev. Cardiol..

[7] Dibben G.O., Dalal H.M., Taylor R.S., Doherty P., Tang L.H., Hillsdon M. (2018). Cardiac rehabilitation and physical activity: Systematic review and meta-analysis. Heart.

[8] Gloeckl R., Schneeberger T., Jarosch I., Kenn K. (2018). Pulmonary rehabilitation and exercise training in chronic obstructive pulmonary disease. Dtsch. ÄRzteblatt Int..

[9] Spruit M.A., Pitta F., McAuley E., ZuWallack R.L., Nici L. (2015). Pulmonary rehabilitation and physical activity in patients with chronic obstructive pulmonary disease. Am. J. Respir. Crit. Care Med..

[10] Dalzell M., Smirnow N., Sateren W., Sintharaphone A., Ibrahim M., Mastroianni L., Zambrano L.V., O’Brien S. (2017). Rehabilitation and exercise oncology program: Translating research into a model of care. Curr. Oncol..

[11] Spence R.R., Heesch K.C., Brown W.J. (2010). Exercise and cancer rehabilitation: A systematic review. Cancer Treat. Rev..

[12] Morrow G.R., Shelke A.R., Roscoe J.A., Hickok J.T., Mustian K. (2005). Management of cancer-related fatigue. Clin. J. Oncol. Nurs..

[13] Dörr W., Engenhart-Cabillic R., Zimmermann J.S. (2002). Normal Tissue Reactions in Radiotherapy and Oncology.

[14] Cup E.H., Pieterse A.J., ten Broek-Pastoor J.M., Munneke M., van Engelen B.G., Hendricks H.T., van der Wilt G.J., Oostendorp R.A. (2007). Exercise Therapy and Other Types of Physical Therapy for Patients With Neuromuscular Diseases: A Systematic Review. Arch. Phys. Med. Rehabil..

[15] Lee Y., Ahn S. (2016). The Effects of Kinesio Taping and Neuromuscular Rehabilitation Exercise for Patients with Acute Whiplash-Associated Disorder. J. Korean Acad. Orthop. Man. Phys. Ther..

[16] Voorn E.L., Koopman F., Nollet F., Brehm M.A. (2019). Aerobic exercise in adult neuromuscular rehabilitation: A survey of healthcare professionals. J. Rehabil. Med..

[17] Frontera W.R. (2003). Exercise and Musculoskeletal Rehabilitation: Restoring Optimal Form and Function. Physician Sportsmed..

[18] Escalante Y., Saavedra J.M., García-Hermoso A., Silva A.J., Barbosa T.M. (2010). Physical exercise and reduction of pain in adults with lower limb osteoarthritis: A systematic review. J. Back Musculoskelet. Rehabil..

[19] American College of Sports Medicine (2013). ACSM’s Health-Related Physical Fitness Assessment Manual.

[20] Warburton D.E., Gledhill N., Quinney A. (2001). Musculoskeletal Fitness and Health. Can. J. Appl. Physiol..

[21] Warburton D.E., Gledhill N., Quinney A. (2001). The effects of changes in musculoskeletal fitness on health. Can. J. Appl. Physiol..

[22] Warburton D.E., McKenzie D.C., Haykowsky M.J., Taylor A., Shoemaker P., Ignaszewski A.P., Chan S.Y. (2005). Effectiveness of high-intensity interval training for the rehabilitation of patients with coronary artery disease. Am. J. Cardiol..

[23] Dun Y., Thomas R.J., Smith J.R., Medina-Inojosa J.R., Squires R.W., Bonikowske A.R., Huang H., Liu S., Olson T.P. (2019). High-intensity interval training improves metabolic syndrome and body composition in outpatient cardiac rehabilitation patients with myocardial infarction. Cardiovasc. Diabetol..

[24] Manley A. (1997). Physical Activity and Health: A Report of the Surgeon General.

[25] Pollock M.L., Gaesser G.A., Butcher J.D., Després J.P., Dishman R.K., Franklin B.A., Garber C.E. (1998). The recommended quantity and quality of exercise for developing and maintaining cardiorespiratory and muscular fitness, and flexibility in healthy adults. Med. Sci. Sport. Exerc..

[26] Andersen L.B., Schnohr P., Schroll M., Hein H.O. (2000). All-Cause Mortality Associated With Physical Activity During Leisure Time, Work, Sports, and Cycling to Work. Arch. Intern. Med..

[27] Schnohr P., Marott J.L., Jensen J.S., Jensen G.B. (2012). Intensity versus duration of cycling, impact on all-cause and coronary heart disease mortality: The Copenhagen City Heart Study. Eur. J. Prev. Cardiol..

[28] Tanasescu M., Leitzmann M.F., Rimm E.B., Willett W.C., Stampfer M.J., Hu F.B. (2002). Exercise type and intensity in relation to coronary heart disease in men. J. Am. Med. Assoc..

[29] Lee I.M., Sesso H.D., Oguma Y., Paffenbarger R.S. (2003). Relative intensity of physical activity and risk of coronary heart disease. Circulation.

[30] Fox E.L., Bartels R.L., Billings C.E., Mathews D.K., Bason R., Webb W.M. (1973). Intensity and distance of interval training programs and changes in aerobic power. Med. Sci. Sport..

[31] Myers J., Prakash M., Froelicher V., Do D., Partington S., Atwood J.E. (2002). Exercise Capacity and Mortality among Men Referred for Exercise Testing. N. Engl. J. Med..

[32] Keteyian S.J., Brawner C.A., Savage P.D., Ehrman J.K., Schairer J., Divine G., Aldred H., Ophaug K., Ades P.A. (2008). Peak aerobic capacity predicts prognosis in patients with coronary heart disease. Am. Heart J..

[33] Rognmo Ø., Hetland E., Helgerud J., Hoff J., Slørdahl S.A. (2004). High intensity aerobic interval exercise is superior to moderate intensity exercise for increasing aerobic capacity in patients with coronary artery disease. Eur. J. Cardiovasc. Prev. Rehabil..

[34] Moholdt T.T., Amundsen B.H., Rustad L.A., Wahba A., Løvø K.T., Gullikstad L.R., Bye A., Skogvoll E., Wisløff U., Slørdahl S.A. (2009). Aerobic interval training versus continuous moderate exercise after coronary artery bypass surgery: A randomized study of cardiovascular effects and quality of life. Am. Heart J..

[35] Kemi O.J., Wisløff U. (2010). High-Intensity Aerobic Exercise Training Improves the Heart in Health and Disease. J. Cardiopulm. Rehabil. Prev..

[36] O’Connor C.M., Whellan D.J., Lee K.L., Keteyian S.J., Cooper L.S., Ellis S.J., Leifer E.S., Kraus W.E., Kitzman D.W., Blumenthal J.A. (2009). Efficacy and safety of exercise training in patients with chronic heart failure HF-ACTION randomized controlled trial. JAMA—J. Am. Med. Assoc..

[37] Cornish A.K., Broadbent S., Cheema B.S. (2011). Interval training for patients with coronary artery disease: A systematic review. Eur. J. Appl. Physiol..

[38] Balady G.J., Williams M.A., Ades P.A., Bittner V., Comoss P., Foody J.A.M., Franklin B., Sanderson B., Southard D. (2007). Core components of cardiac rehabilitation/secondary prevention programs: 2007 update—A sci. statement from the Am. Heart Assoc. exercise, cardiac rehabilitation, and prevention comm., the council on clinical cardiology; the councils on cardiovascular nu. Circulation.

[39] Kobashigawa J.A., Leaf D.A., Lee N., Gleeson M.P., Liu H., Hamilton M.A., Moriguchi J.D., Kawata N., Einhorn K., Herlihy E. (1999). A controlled trial of exercise rehabilitation after heart transplantation. N. Engl. J. Med..

[40] Bohannon R.W. (1995). Sit-to-stand test for measuring performance of lower extremity muscles. Percept. Mot. Ski..

[41] Bohanno R.W. (2011). Test-retest reliability of the five-repetition sit-to-stand test: A systematic review of the literature involving adults. J. Strength Cond. Res..

[42] Jiménez C.R., Bennett P., García A.O., Cuesta Vargas A.I. (2019). Fatigue detection during sit-to-stand test based on surface electromyography and acceleration: A case study. Sensors.

[43] Shephard R. (2001). Absolute versus relative intensity of physical activity in a dose-response context. Med. Sci. Sport..

[44] Ainsworth B., Haskell W.L., Leon A.S., Jacobs D.R., Montoye H.J., Sallis J.F., Paffenbarger R.S. (1993). Compendium of physical activities: Classification of energy costs of human physical activities. Med. Sci. Sport. Exerc..

[45] Schutz Y., Weinsier R.L., Hunter G.R. (2001). Assessment of free-living physical activity in humans: An overview of currently available and proposed new measures. Obes. Res..

[46] Ainsworth B.E., Haskell W.L., Whitt M.C., Irwin M.L., Swartz A.M., Strath S.J., O’brien W.L., Bassett D.R., Schmitz K.H., Emplaincourt P. (2000). Compendium of Physical Activities: An update of activity codes and MET intensities. Med. Sci. Sport. Exerc..

[47] Savage P.D., Toth M.J., Ades P.A. (2007). A re-examination of the metabolic equivalent concept in individuals with coronary heart disease. J. Cardiopulm. Rehabil. Prev..

[48] Qi J., Yang P., Waraich A., Deng Z., Zhao Y., Yang Y. (2018). Examining sensor-based physical activity recognition and monitoring for healthcare using Internet of Things: A systematic review. J. Biomed. Inform..

[49] Zeni A.I., Hoffman M.D., Clifford P.S. (1996). Relationships among heart rate, lactate concentration, and perceived effort for different types of rhythmic exercise in women. Arch. Phys. Med. Rehabil..

[50] da Cunha F.A., Farinatti P.d.T.V., Midgley A.W. (2011). Methodological and practical application issues in exercise prescription using the heart rate reserve and oxygen uptake reserve methods. J. Sci. Med. Sport.

[51] Reybrouck T., Mertens L., Brusselle S., Weymans M., Eyskens B., Defoor J., Gewillig M. (2000). Oxygen uptake versus exercise intensity: A new concept in assessing cardiovascular exercise function in patients with congenital heart disease. Heart.

[52] Jette M., Sidney K., Blümchen G. (1990). Metabolic equivalents (METS) in exercise testing, exercise prescription, and evaluation of functional capacity. Clin. Cardiol..

[53] Fukuda K., Straus S.E., Hickie I., Sharpe M.C., Dobbins J.G., Komaroff A. (1994). The chronic fatigue syndrome: A comprehensive approach to its definition and study. Ann. Intern. Med..

[54] Dittner A.J., Wessely S.C., Brown R.G. (2004). The assessment of fatigue: A practical guide for clinicians and researchers. J. Psychosom. Res..

[55] Abd-Elfattah H.M., Abdelazeim F.H., Elshennawy S. (2015). Physical and cognitive consequences of fatigue: A review. J. Adv. Res..

[56] Karthick P.A., Ghosh D.M., Ramakrishnan S. (2018). Surface electromyography based muscle fatigue detection using high-resolution time-frequency methods and machine learning algorithms. Comput. Methods Programs Biomed..

[57] Subasi A., Kiymik M.K. (2010). Muscle fatigue detection in EMG using time-frequency methods, ICA and neural networks. J. Med. Syst..

[58] Al-Mulla M.R., Sepulveda F., Colley M. (2011). A Review of Non-Invasive Techniques to Detect and Predict Localised Muscle Fatigue. Sensors.

[59] Stoykov N.S., Lowery M.M., Kuiken T.A. (2005). A finite-element analysis of the effect of muscle insulation and shielding on the surface EMG signal. IEEE Trans. Biomed. Eng..

[60] Annett J. (2002). Subjective rating scales: Science or art?. Ergonomics.

[61] Borg G. (2001). Borg’s range model and scales. Int. J. Sport Psychol..

[62] Zamunér A.R., Moreno M.A., Camargo T.M., Graetz J.P., Rebelo A.C., Tamburús N.Y., da Silva E. (2011). Assessment of subjective perceived exertion at the anaerobic threshold with the Borg CR-10 scale. J. Sport. Sci. Med..

[63] Paillard T. (2012). Effects of general and local fatigue on postural control: A review. Neurosci. Biobehav. Rev..

[64] Roldán-Jiménez C., Bennett P., Cuesta-Vargas A.I. (2015). Muscular activity and fatigue in lower-limb and trunk muscles during different sit-to-stand tests. PLoS ONE.

[65] Aguirre A., Casas J., Céspedes N., Múnera M., Rincon-Roncancio M., Cuesta-Vargas A., Cifuentes C.A. Feasibility study: Towards Estimation of Fatigue Level in Robot-Assisted Exercise for Cardiac Rehabilitation. Proceedings of the 2019 IEEE 16th International Conference on Rehabilitation Robotics (ICORR).

[66] Mokaya F., Lucas R., Noh H.Y., Zhang P. Burnout: A wearable system for unobtrusive skeletal muscle fatigue estimation. Proceedings of the 2016 15th ACM/IEEE International Conference on Information Processing in Sensor Networks (IPSN).

[67] Camomilla V., Bergamini E., Fantozzi S., Vannozzi G. (2018). Trends supporting the in-field use of wearable inertial sensors for sport performance evaluation: A systematic review. Sensors.

[68] Ejupi A., Gschwind Y.J., Valenzuela T., Lord S.R., Delbaere K. (2016). A kinect and inertial sensor-based system for the self-assessment of fall risk: A home-based study in older people. Hum. Comput. Interact..

[69] McGinnis R.S., Cain S.M., Davidson S.P., Vitali R.V., Perkins N.C., McLean S.G. (2016). Quantifying the effects of load carriage and fatigue under load on sacral kinematics during countermovement vertical jump with IMU-based method. Sport. Eng..

[70] Zhang J., Lockhart T.E., Soangra R. (2014). Classifying lower extremity muscle fatigue during walking using machine learning and inertial sensors. Ann. Biomed. Eng..

[71] Hollander J.E., Carr B.G. (2020). Virtually perfect? Telemedicine for COVID-19. N. Engl. J. Med..

[72] Jakicic J., Otto A.D. (2005). Physical activity considerations for the treatment and prevention of obesity. Am. J. Clin. Nutr..

[73] Hsiao M.Y., Li C.M., Lu I.S., Lin Y.H., Wang T.G., Han D.S. (2018). An investigation of the use of the Kinect system as a measure of dynamic balance and forward reach in the elderly. Clin. Rehabil..

[74] Kakria P., Tripathi N., Kitipawang P. (2015). A real-time health monitoring system for remote cardiac patients using smartphone and wearable sensors. Int. J. Telemed. Appl..

[75] Moohialdin A.S., Suhariadi B.T., Siddiqui M.K. Practical validation measurements of a physiological status monitoring sensor in real construction activities. Proceedings of the Streamlining Information Transfer between Construction and Structural Engineering.

[76] Kim J.H., Roberge R., Powell J., Shafer A., Williams W.J. (2013). Measurement accuracy of heart rate and respiratory rate during graded exercise and sustained exercise in the heat using the Zephyr BioHarness™. Int. J. Sport. Med..

[77] American College of Sports Medicine (2012). ACSM’s Resource Manual for Guidelines for Exercise Testing and Prescription.

[78] Swain D.P., Brawner C.A., American College of Sports Medicine (2014). ACSM’s Resource Manual for Guidelines for Exercise Testing and Prescription.

[79] Arney B., Glover R., Fusco A., Cortis C., de Koning J., Erp T., Jaime S., Mikat R., Porcari J., Foster C. (2019). Comparison of rating of perceived exertion scales during incremental and interval exercise. Kinesiology.

[80] Colado J.C., Brasil R.M. (2019). Concurrent and Construct Validation of a Scale for Rating Perceived Exertion in Aquatic Cycling for Young Men. J. Sport. Sci. Med..

[81] Lessley D., Crandall J., Shaw G., Kent R., Funk J. (2004). A Normalization Technique for Developing Corridors from Individual Subject Responses.

[82] Moorhouse K. An improved normalization methodology for developing mean human response curves. Proceedings of the International Technical Conference on the Enhanced Safety of Vehicles.

[83] Yoganandan N., Arun M.W., Pintar F.A. (2014). Normalizing and scaling of data to derive human response corridors from impact tests. J. Biomech..

[84] Skiena S.S. (2017). The Data Science Design Manual.

[85] Berrar D. (2019). Cross-validation. Encycl. Bioinform. Comput. Biol..

[86] Pedregosa F., Varoquaux G., Gramfort A., Michel V., Thirion B., Grisel O., Blondel M., Prettenhofer P., Weiss R., Dubourg V. (2011). Scikit-learn: Machine learning in Python. J. Mach. Learn. Res..

[87] Algamal Z.Y., Lee M.H. (2015). Penalized logistic regression with the adaptive LASSO for gene selection in high-dimensional cancer classification. Expert Syst. Appl..

[88] Maman Z.S., Yazdi M.A.A., Cavuoto L.A., Megahed F.M. (2017). A data-driven approach to modeling physical fatigue in the workplace using wearable sensors. Appl. Ergon..

[89] Afsar P., Cortez P., Santos H. (2015). Automatic visual detection of human behavior: A review from 2000 to 2014. Expert Syst. Appl..

[90] Ghaderyan P., Abbasi A., Saber S. (2018). A new algorithm for kinematic analysis of handwriting data; towards a reliable handwriting-based tool for early detection of alzheimer’s disease. Expert Syst. Appl..

[91] Rescio G., Leone A., Siciliano P. (2018). Supervised machine learning scheme for electromyography-based pre-fall detection system. Expert Syst. Appl..

[92] Ryu J., Kim D.H. (2017). Real-time gait subphase detection using an EMG signal graph matching (ESGM) algorithm based on EMG signals. Expert Syst. Appl..

[93] Yigit H. A weighting approach for KNN classifier. Proceedings of the 2013 International Conference on Electronics, Computer and Computation (ICECCO).

[94] Erickson B.J., Korfiatis P., Akkus Z., Kline T.L. (2017). Machine learning for medical imaging. Radiographics.

[95] Madzarov G., Gjorgjevikj D., Chorbev I. (2009). A multi-class SVM classifier utilizing binary decision tree. Informatica.

[96] Mahmon N.A., Ya’acob N. A review on classification of satellite image using Artificial Neural Network (ANN). Proceedings of the 2014 IEEE 5th Control and System Graduate Research Colloquium.

[97] Breiman L. (2001). Random forests. Mach. Learn..

[98] Dietterich T.G. (2000). Ensemble methods in machine learning. International Workshop on Multiple Classifier Systems.

[99] Maman Z.S., Chen Y.J., Baghdadi A., Lombardo S., Cavuoto L.A., Megahed F.M. (2020). A data analytic framework for physical fatigue management using wearable sensors. Expert Syst. Appl..

[100] McInnes L., Healy J., Melville J. (2018). Umap: Uniform manifold approximation and projection for dimension reduction. arXiv.

[101] Strassmann A., Steurer-Stey C., Dalla Lana K., Zoller M., Turk A.J., Suter P., Puhan M.A. (2013). Population-based reference values for the 1-min sit-to-stand test. Int. J. Public Health.

[102] Parkinson S., Campbell A., Dankaerts W., Burnett A., O’Sullivan P. (2013). Upper and lower lumbar segments move differently during sit-to-stand. Man. Ther..

